# Experience-dependent plasticity of multiple receptive field properties in lateral geniculate binocular neurons during the critical period

**DOI:** 10.3389/fncel.2025.1574505

**Published:** 2025-04-28

**Authors:** Meng Pan, Jingjing Ye, Yijing Yan, Ailin Chen, Xinyu Li, Xin Jiang, Wei Wang, Xin Meng, Shujian Chen, Yu Gu, Xuefeng Shi

**Affiliations:** ^1^Tianjin Key Laboratory of Ophthalmology and Visual Science, Tianjin Eye Institute, Tianjin Eye Hospital, Clinical College of Ophthalmology, Tianjin Medical University, Tianjin, China; ^2^School of Medicine, Nankai University, Tianjin, China; ^3^State Key Laboratory of Medical Neurobiology and MOE Frontiers Center for Brain Science, Institutes of Brain Science, Fudan University, Shanghai, China

**Keywords:** dorsolateral geniculate nucleus, ocular dominance plasticity, direction selectivity, orientation selectivity, monocular deprivation, inhibitory circuitry

## Abstract

The visual thalamus serves as a critical hub for feature preprocessing in visual processing pathways. Emerging evidence demonstrates that experience-dependent plasticity can be revealed by monocular deprivation (MD) in the dorsolateral geniculate nucleus (dLGN) of the thalamus. However, whether and how this thalamic plasticity induces changes in multiple receptive field properties and the potential mechanisms remain unclear. Using *in vivo* electrophysiology, here we show that binocular neurons in the dLGN of 4-day MD mice starting at P28 undergo a significant ocular dominance (OD) shift during the critical period. This OD plasticity could be attributed to the potentiation of ipsilateral eye responses but not to the depression of deprived eye responses, contrasting with conventional observations in the primary visual cortex (V1). The direction and orientation selectivity of ipsilateral eye responses, but not of contralateral eye responses in these neurons, were dramatically reduced. Developmental analysis revealed pre-critical and critical period-associated changes in densities of both GABA positive neurons and GABA_A_ receptor α1 subunit (GABRA1) positive neurons. However, early compensatory inhibition from V1 feedback in P18 MD mice maintained network stability with no changes in OD and feature selectivity. Mechanistically, pharmacological activation of GABA_A_ receptors rescued the MD-induced OD shifts and feature selectivity impairments in critical period MD mice, operating independently of the V1 feedback. Furthermore, under different contrast levels and spatial frequencies, these critical period-associated changes in receptive field properties still indicate alterations in ipsilateral eye responses alone. Together, these findings provide novel insights into the developmental mechanisms of thalamic sensory processing, highlighting the thalamus as an active participant in experience-dependent visual plasticity rather than merely a passive relay station. The identified GABA-mediated plasticity mechanisms offer potential therapeutic targets for visual system disorders.

## 1 Introduction

The mammalian visual system employs a canonical hierarchical pathway originating in retinal circuits, which relay visual signals through the dorsolateral geniculate nucleus (dLGN) of the thalamus before projecting to lamina-specific targets in the primary visual cortex (V1) (Litvina and Chen, 2017; Bauer et al., 2021). Neurons at various stages of this pathway integrate visual information and generate responses selectively, such as responses to stimuli with specific directions or orientations (Douglas and Martin, 2004; Vaney et al., 2012; Priebe and Ferster, 2012). This feature processing helps to give rise to visual perception and behavior essential for survival (Livingstone and Hubel, 1988). Feature preprocessing in the visual thalamus is crucial for visual processing and perception (Allen and Freeman, 2006; Krahe et al., 2011; Piscopo et al., 2013).

Neural circuitry in mammalian visual regions develops through interactions with the environment (Hooks and Chen, 2020). Studies have demonstrated that the neural circuits in V1 exhibit significant experience-dependent reorganization during early postnatal life, known as “critical period,” and the maturation and fine regulation of this circuity rely on experience-dependent processes at this stage (Ko et al., 2013; Voss, 2013). Abnormal visual experience during the critical period will induce the disruption of binocular vision and abnormal development of visual functions (Hensch, 2005), a condition clinically termed amblyopia.

Since Hubel and Wiesel (1962) first proposed the physiological mechanism of ocular dominance (OD) plasticity in V1, experience-dependent plasticity of V1 during the critical period has been extensively studied. Four-day monocular deprivation (MD) has become one of the most classic animal models for understanding the OD plasticity (Hubel and Wiesel, 1970; Gordon and Stryker, 1996). However, recent research has challenged the view that the visual cortex is the sole site of critical-period visual plasticity (Gilbert et al., 2009; Gilbert and Wiesel, 1992) and demonstrated that subcortical areas, including the superior colliculus (Hu et al., 2024) and dLGN (Sommeijer et al., 2017; Huh et al., 2020; Li et al., 2023), also exhibit such plasticity. Emerging evidence also suggests that neurons in the dLGN can receive binocular inputs (Rompani et al., 2017; Jaepel et al., 2017), challenging the previous view that dLGN neurons only receive inputs exclusively from the contralateral or ipsilateral eye (Casagrande and Boyd, 1996). dLGN exhibits binocular integration and OD plasticity, similar to that observed in V1 (Howarth et al., 2014; Zeater et al., 2015; Li and Gu, 2020; Sommeijer et al., 2017; Rose and Bonhoeffer, 2018).

The role of visual experience in shaping thalamic function and circuitry is increasingly being recognized. However, whether and how this thalamic plasticity induces changes in multiple receptive field properties and the potential mechanisms remain poorly understood. In V1, changes in inhibitory circuits have been identified as key mechanisms for critical-period plasticity, with the balance between excitation and inhibition in specific neural circuits being crucial for V1 plasticity (Hensch and Fagiolini, 2005; Fagiolini et al., 2004). A recent study has shown that inhibitory neural circuits play an important role in regulating experience-dependent plasticity in the dLGN (Sommeijer et al., 2017). Additionally, dLGN neurons receive V1 feedback from layer 6 neurons through direct excitatory projection as well as indirect inhibitory projection via thalamic reticular nucleus (TRN) and local interneurons (Thompson et al., 2016); thus responses of dLGN neurons may be altered by the V1 feedback (Denman and Contreras, 2015; Kirchgessner et al., 2020). However, whether the intra-thalamic inhibitory circuits and V1 feedback are involved in the alterations of multiple receptive field properties following MD remains unclear.

Here, we hypothesize that a 4-day MD during the critical period alters both OD and feature selectivity in dLGN binocular neurons, mediated by intra-thalamic inhibitory circuits involving GABAergic signaling. With *in vivo* electrophysiology, we show that in 4-day MD mice, dLGN binocular neurons exhibit significant OD shifts during the critical period, driven by potentiation of ipsilateral eye responses, unlike in V1. This is accompanied by reduced direction and orientation selectivity for the ipsilateral eye, with no change for the contralateral eye. Developmental analysis reveals pre-critical and critical period- associated changes in the densities of both GABA positive neurons and GABA_A_ receptor α1 subunit (GABRA1) positive neurons. Pharmacological activation of GABA_A_ receptor rescued MD-induced OD shifts and feature selectivity impairments in critical period MD mice, operating independently of the V1 feedback. Further, these critical period-associated effects are consistent across different contrast levels and spatial frequencies (SFs). Together, these findings highlight the thalamus as an active participant in experience-dependent visual plasticity and suggest GABA-mediated mechanisms as potential therapeutic targets for visual disorders.

## 2 Materials and methods

### 2.1 Animals

Specific pathogen-free (SPF) male C57BL/6J mice purchased from Beijing Vital River Laboratory Animal Technology Co., Ltd. (Beijing, China) were used in this study, aged postnatal day (P)13 to P64. Mice were housed in groups of five per cage under standard conditions with a 12 h/12 h light/dark cycle, constant temperature (22 ± 1°C), and relative humidity (45 ± 5%). Mice were allowed free access to standard food and water. The experimental procedures were approved by the Institutional Animal Care and Use Committee of Tianjin Medical University.

### 2.2 Monocular deprivation

Monocular deprivation (MD) was performed by suturing the right eyelid under anesthesia. Tobramycin-dexamethasone ointment was applied to prevent infection. Anesthetized mice were recovered on a heating pad to maintain their temperature at 37°C through a feedback heater control module (Frederick Haer Company, Bowdoinham, ME, Unites States) until they were alert and mobile and then returned to their home cage. The durations of MD were 2 days, 4 days and 7 days in this experiment. Sutures were checked daily to ensure closure and uninfected. Prior to *in vivo* electrophysiological recordings, the eyelid suture was carefully removed using sterile surgical scissors to reopen the right eye. Animals exhibiting eyelid abnormalities or ocular damage (e.g., corneal opacity or mechanical injury) during the reopening procedure were excluded from subsequent experiments.

### 2.3 Surgery

The anesthetized mice were fixed to a customized stereotaxic surgical stage and placed on a heating pad to maintain their temperature at 37°C. To prevent corneal desiccation and cataract formation, sterile ophthalmic lubricant was applied to both eyes, followed by the application of light-occluding tape. Mice with MD were examined after suture removal before surgery and recording. The scalp was carefully shaved and excised to expose the underlying skull and cranial sutures. A custom metal head ring and two cranial nails were affixed with dental acrylic to the skull of the left hemisphere, ensuring precise alignment of the dLGN within the ring center while minimizing mechanical damage to the underlying tissue.

A small craniotomy above dLGN (2.2–2.7 mm posterior from bregma and 2.0–2.5 mm lateral from midline) was made to expose the brain. Overlying tissue, including the cortex and hippocampus above the dLGN, was meticulously aspirated via a vacuum suction device. For cortical instant inactivation, we made the second craniotomy ipsilateral to the hemisphere of recorded dLGN to expose the whole V1 (1.5–3.5 mm lateral from the midline and 0.3–0.8 mm anterior from the lambda suture). Throughout the procedure, the exposed brain was kept moist with artificial cerebrospinal fluid (ACSF).

### 2.4 *In vivo* electrophysiology

After craniotomy, mice were positioned under a stereomicroscope for electrophysiological recordings. Reference electrodes were fixed to the cranial nails. A 16-channel silicon probe (ASSY-1-16-1, Lotus Biochips, United States) was gradually advanced until positioned just above the dLGN and then carefully inserted into the pial surface. Recordings commenced once all 16 channels were confirmed to span the dLGN and exhibited robust visual response signals. For each animal, recordings were performed at 2–3 distinct sites within the dLGN. In the subset of cortical instant inactivation, V1 was also targeted to recording before and after using muscimol. Neural signals were acquired using the OmniPlex Neural Recording Data Acquisition System (Plexon Inc., Dallas, United States). To isolate monocular responses, an opaque occluder was placed over one eye while recording responses to the other eye stimulation. Throughout the recording, the exposed brain was kept moist with ACSF, and the body temperature was maintained at 37°C using a feedback-controlled heating system. Anesthesia depth was monitored via the toe-pinch reflex, with supplemental urethane (5%) administered as needed. The recording probes were coated in 1,10-dioctadecyl-3,3,30,30-tetramethylindocarbocyanine percolate (DiI, Thermo Fisher Scientific, D282). Following recordings, mice were transcardially perfused, and the brains were sectioned for histological verification of electrode placement.

### 2.5 Visual stimuli

Visual stimuli were generated using Psychopy v3.0 and displayed on an LCD monitor (37.5 cm × 30 cm, 60 Hz refresh rate, ~50 cd/m^2^ mean luminance) positioned 20 cm in front of the animal’s eyes. The stimuli consisted of drifting sinusoidal gratings with varying orientations, spatial frequencies, and contrasts. Gratings drifted perpendicular to their orientation, with directions varied between 0° and 330° (12 steps at 30° spacing). Stimulus contrasts were set at 100% (1.0), 60% (0.6), and 30% (0.3). The spatial frequencies were set at 0.02, 0.05 and 0.08 cycles/degree. Temporal frequency was fixed at 2 Hz. The default parameters for optimal contrast and spatial frequency were set to 1.0 and 0.05 cycles/degree, respectively. Each stimulus was presented for 2 s, followed by a 1-s inter-stimulus interval displaying a uniform gray screen to measure spontaneous firing rates. For each recording site, the stimulus sequence was repeated eight times in a pseudorandom order, for the contralateral and ipsilateral eyes, respectively.

### 2.6 Immunofluorescence staining

Anesthetized animals were transcardially perfused first with ice-cold saline followed by ice-cold 4% paraformaldehyde (PFA, Biotopped, China). The brains were carefully dissected and post-fixed in 4%PFA at 4°C for 24 h. Coronal sections (100 μm thick) were prepared using a Leica VT 1000S vibratome (Leica, Germany) in a chilled phosphate-buffered saline (PBS) bath. Slices were transferred to 24-well plates, washed three times in PBS for 5 min each, and incubated in blocking solution [5% bovine serum albumin (BSA) + 0.3% Triton X-100 in PBS] for 2 h at room temperature. For immunostaining, slices were incubated overnight at 4°C with primary antibodies diluted in PBS: mouse anti-GABA (1:500, Sigma-Aldrich, Cat# A0310) and rabbit anti-GABRA1 (1:200, Proteintech, Cat# 12410-1-AP). After three washes, slices were incubated for 2 h at room temperature in the dark with secondary antibodies: anti-mouse IgG conjugated to Alexa Fluor 647 (1:200, Abcam, Cat # ab150115) and anti-rabbit IgG conjugated to Alexa Fluor 594 (1:200, Abcam, Cat # ab150080). After washing 3 times, slices were stained with DAPI (1:100, Sigma-Aldrich, Cat# 32670) working solution for 8 min and washed for 3 times. Slices were mounted on glass slides, and fluorescent images were acquired using a Leica SP8 SR confocal microscope. For quantitative analysis, ImageJ software (NIH) was used to process 10-plane z-stack maximum intensity projections of images from three slices per animal. Cell counts were performed manually to ensure accuracy. For analysis of the immunofluorescence images, we firstly determined a threshold which can be used to effectively identify the cells co-labeled with GABA/GABRA1 and DAPI in a subset of images. In our final counting and statistical analysis, we only included neurons with fluorescence intensity above this threshold and manually checked. Further, to determine whether apoptosis was occurring, we carried out Tunel staining on the dLGN in both P32 Ctrl and MD mice.

### 2.7 Pharmacological manipulation

To activate intra-thalamic inhibitory circuits, the GABA_A_ receptor (GABRA) agonist, muscimol hydrobromide (10 μL, 0.1 mM in ACSF, Sigma-Aldrich, Cat# G019-5MG, China), was applied to the surface of the dLGN. Electrophysiological recordings were performed in the same animal both before and after muscimol administration. To instantly inactivate V1 in P18 MD and P32 MD mice, we topical applied the muscimol (10 μL, 5 mM in ACSF, Sigma-Aldrich, Cat# G019-5MG, China) to the surgically exposed surface of V1. After 10 min, the visual response abolished in all cortical layers of V1.

### 2.8 Spike detection, sorting, and electrophysiological data analysis

Neuronal activity data were exported and preprocessed using an offline analysis software (OmniPlex v4.0, Plexon Inc., Dallas, TX, United States). Subsequent analyses, including spike detection, spike sorting, and electrophysiological parameter quantification, were performed using custom MATLAB scripts (MathWorks, https://www.mathworks.com/). Detailed methodologies for these analyses have been described in our previous works (Hu et al., 2024; Hao et al., 2022; Shi et al., 2017; Shi et al., 2018).

To quantify OD, the ocular dominance index (ODI) was used to assess the binocularity of the dLGN neurons and was calculated according to the following formula: ODI = (C − I)/(C + I), where C and I represent the response magnitudes to visual stimuli presented to the contralateral and ipsilateral eyes, respectively.

Additionally, dLGN neurons were assigned OD scores according to the methods of the Hubel and Wiesel (1962) classification system, and the contralateral bias index (CBI) was then calculated according to the following formula: CBI = [(*N*1–*N*7) + (2/3) * (*N*2–*N*6) + (1/3) * (*N*3–*N*5) + *N*]/2*N*, where *Nx* represents the number of neurons assigned to OD category *x*, and *N* denotes the total number of neurons analyzed.

To assess the degree of direction selectivity (DS) and orientation selectivity (OS), we computed a global direction selectivity index (gDSI) and a global orientation selectivity index (gOSI). The gDSI and gOSI were derived from the vector sum of responses normalized by the scalar sum of responses across 12 directions or 6 orientations, respectively (Shi et al., 2017, 2018). Specifically, the gDSI and gOSI were calculated using the following formulas: $\mathrm{gDSI}=|\frac{\sum R_{\theta }e^{i\theta }}{\sum R_{\theta }}|$ , $\mathrm{gOSI}=|\frac{\sum R_{\theta }e^{i2\theta }}{\sum R_{\theta }}|$ , where *R_θ_* represents the response magnitude of spikes at *θ* direction of drifting gratings.

### 2.9 Statistical analysis

Statistical analyses were conducted using GraphPad Prism software (version 9.1.1). Mann–Whitney *U* tests were applied for comparisons between two independent, non-normally groups. The Kruskal–Wallis test was used for comparisons involving more than two non-normally distributed groups, followed by post-hoc multiple comparisons with Bonferroni corrections when significant differences (*p* < 0.05) were detected. Parametric comparisons between two independent groups were performed using the unpaired Student’s *t*-test, while the paired Student’s *t*-test was used for within-subject comparisons of two measurements. Data are presented as mean ± standard error of the mean (SEM), with statistical significance set at *p* < 0.05. Significance levels are denoted as follows: ^*^p < 0.05, ^**^p < 0.01, and ^***^p < 0.001 applicable to all figures.

## 3 Results

### 3.1 Four-day MD during the critical period induced OD shifts in dLGN neurons driven by ipsilateral potentiation

**Figure 1:**
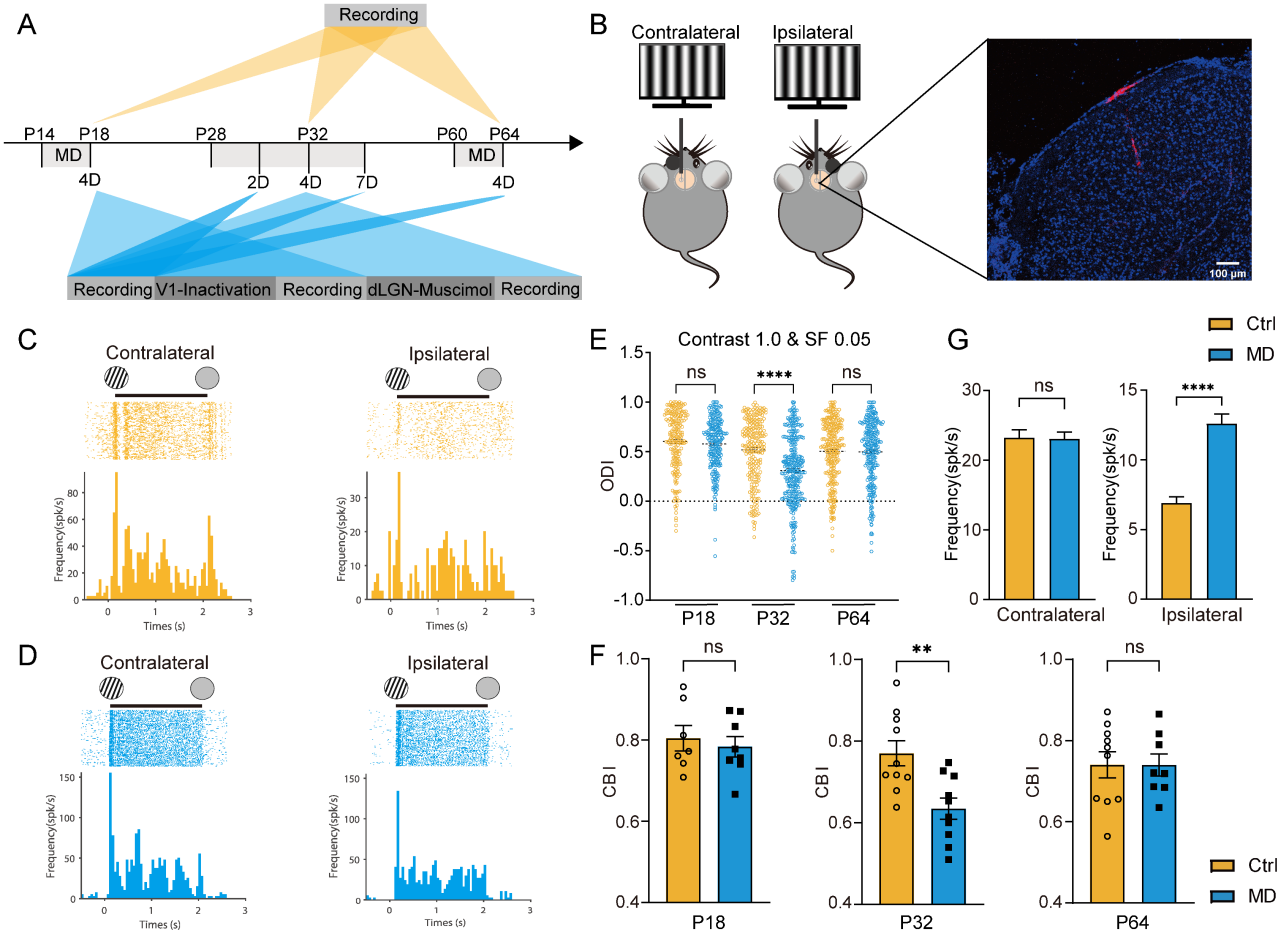
Four-day MD during the critical period induced OD shifts in dLGN neurons via the potentiation of ipsilateral-eye responses. **(A)** Experimental timeline. MD, monocular deprivation; 2D, 2-day MD; 4D, 4-day MD; 7D, 7-day MD; V1-inactivation, instant V1 inactivation using muscimol (5 mM); dLGN-muscimol, pharmacological activation of GABA_A_ receptors via muscimol (0.1 mM). **(B)** Left, experimental setup. Right, a representative image showing trace of multichannel recording probe (red) in a coronal slice containing dLGN stained by DAPI (blue). Scale bar, 100 μm. **(C,D)** Examples of spike raster plots (for all directions) and peristimulus time histograms (PSTHs, for all direction) of a dLGN neuron in control **(B)** and MD group **(C)** in response to full-field drifting grating to contralateral (left) and ipsilateral eye (right). Solid lines indicate 0–2 s after the onset of drifting grating stimulation. **(E)** Comparisons of ODI for recorded dLGN neurons in P18, P32, and P64 between control (yellow) and MD group (blue). In each plot, each point represents the ODI of a single dLGN neurons (Kruskal–Wallis test, P18 Ctrl, *n* = 203 cells from 7 mice; P18 MD, *n* = 238 cells from 8 mice; P32 Ctrl, *n* = 220 cells from 10 mice; P32 MD, *n* = 318 cells from 10 mice; P64 Ctrl, *n* = 271 cells from 10 mice; P64 MD, *n* = 264 cells from 8 mice; respectively). ^ns^*p* > 0.05 and ^****^p < 0.0001. **(F)** Comparisons of CBI between control and MD group for P18 (left), P32 (middle), and P64 mice (right). Open circles and solid square represent individual CBIs for each Ctrl and MD mice. Unpaired *t*-test, two-tailed. ^ns^*p* > 0.05 and ^**^p < 0.01. Error bars represent mean ± SEM. **(G)** Comparisons of mean responses of contralateral (left) and ipsilateral eye (*p* < 0.0001) in dLGN neurons between P32 Ctrl and P32 MD mice; Mann–Whitney *U* test. ^ns^*p* > 0.05 and ^****^p < 0.0001.

Previous studies have demonstrated that long-term MD can induce OD shifts in dLGN neurons, both during juvenile development and in adulthood (Jaepel et al., 2017; Sommeijer et al., 2017). To confirm whether and when there exists a short-term MD induced V1-like OD shift in the dLGN and how the OD shift occurs, we performed *in vivo* multichannel recordings using 16 channels silicon probe targeting the left dLGN in both control and 4-day MD groups. Recordings were conducted during three developmental stages: the critical period (CP) (P28–P32), adulthood (P60–P64), and pre-critical period (pre-CP) (P14–P18). Visual stimuli consisted of 12-direction drifting gratings presented at optimal contrast (1.0) and spatial frequency (0.05 cycles/degree) to the contralateral and ipsilateral eyes. Neuronal responses were recorded (Figures 1A–D), and ODI and CBI representing the degree of ocular dominance at the cellular and animal levels, respectively, were calculated.

Consistent with our previous findings (Li et al., 2023), a significant OD shift toward the ipsilateral eye was observed following 4-day MD in dLGN, but only during the critical period (Ctrl vs. MD for ODI: pre-CP: *p* = 0.6032, CP: *p* < 0.0001, adulthood: *p* > 0.9999, Figure 1E; for CBI: pre-CP: *p* = 0.6113, CP: *p* = 0.0033, adulthood: *p* = 0.9934, Figure 1F). In V1, OD plasticity is known to involve two distinct mechanistic components (Frenkel and Bear, 2004): (1) a rapid loss of deprived-eye responses following short-term MD, which is characteristic of critical-period plasticity and regulated by cortical inhibitory circuits, and (2) a gradual potentiation of non-deprived-eye responses following prolonged MD, which also occur in adult V1. Interestingly, the OD shift induced by 4-day MD during the CP in dLGN was driven exclusively by potentiation of ipsilateral eye responses (Ctrl vs. MD: *p* < 0.0001), rather than depression of deprived-eye responses (Ctrl vs. MD: *p* = 0.6981, Figure 1G), contrasting sharply with the mechanisms observed in V1 (Frenkel and Bear, 2004). These findings suggest that visual experience is essential for the development of binocular integration in dLGN neurons during the CP. Furthermore, the mechanisms underlying experience-dependent plasticity in the dLGN appear to differ fundamentally from those in V1.

Additionally, it remains unclear whether different classes of neurons manifest different OD plasticity in the dLGN. To address this, we classified recorded dLGN neurons into narrow-spiking and broad-spiking cells based on their action potential waveforms (Hao et al., 2022) as narrow-spiking units are widely recognized as inhibitory neurons, while broad-spiking units are considered excitatory. Further analysis revealed that both excitatory and inhibitory neuronal populations displayed OD shifts following 4-day MD during the critical period (Supplementary Figures S1A–C). These shifts were both mediated by enhanced ipsilateral eye responses (Supplementary Figures S1D,E), suggesting that excitatory and inhibitory neurons in the dLGN may engage shared pathways to drive OD plasticity.

OD shifts in V1 is known to involve a transition from depression of the deprived-eye responses to potentiation of the non-deprived-eye responses following a prolonged MD (Frenkel and Bear, 2004). It remains unclear whether the dLGN also undergoes dynamic changes in response to varying MD durations. To further explore this question, we performed *in vivo* multichannel recordings targeting the left dLGN in 2-day MD and 7-day MD groups, respectively. Consistent with a previous study (Sommeijer et al., 2017), we observed gradually shifted OD toward the ipsilateral eye as MD duration increased (Supplementary Figures S2A,B). The contralateral-eye responses exhibited an initial increase (2-day MD) followed by a subsequent decrease in 7-day MD mice (Supplementary Figure S2C). Since the excitatory input from the deprived eye cannot increase following either short-term or long-term deprivation, the sole plausible explanations for the increased deprived-eye responses in 2-day MD mice should be the decreased inhibition or homeostatic response in dLGN, which differed significantly from the loss of deprived eye responses in V1 during early MD. If intra-thalamic inhibition is reduced but not enhanced following MD, the delayed emergence of deprived-eye response suppression (observed only after 7-day MD) suggests that excitatory input from the deprived eye undergoes only weak or nonsignificant alterations during early MD. In contrast, the ipsilateral-eye responses showed a continuous increase starting at early MD with increasing MD duration (Supplementary Figure S2C). The potential role of intra-thalamic inhibition in mediating early MD-induced ocular dominance shifts and ipsilateral response potentiation requires further investigation.

### 3.2 Four-day MD during the critical period reduced direction and orientation selectivity of ipsilateral eye responses in dLGN neurons

**Figure 2:**
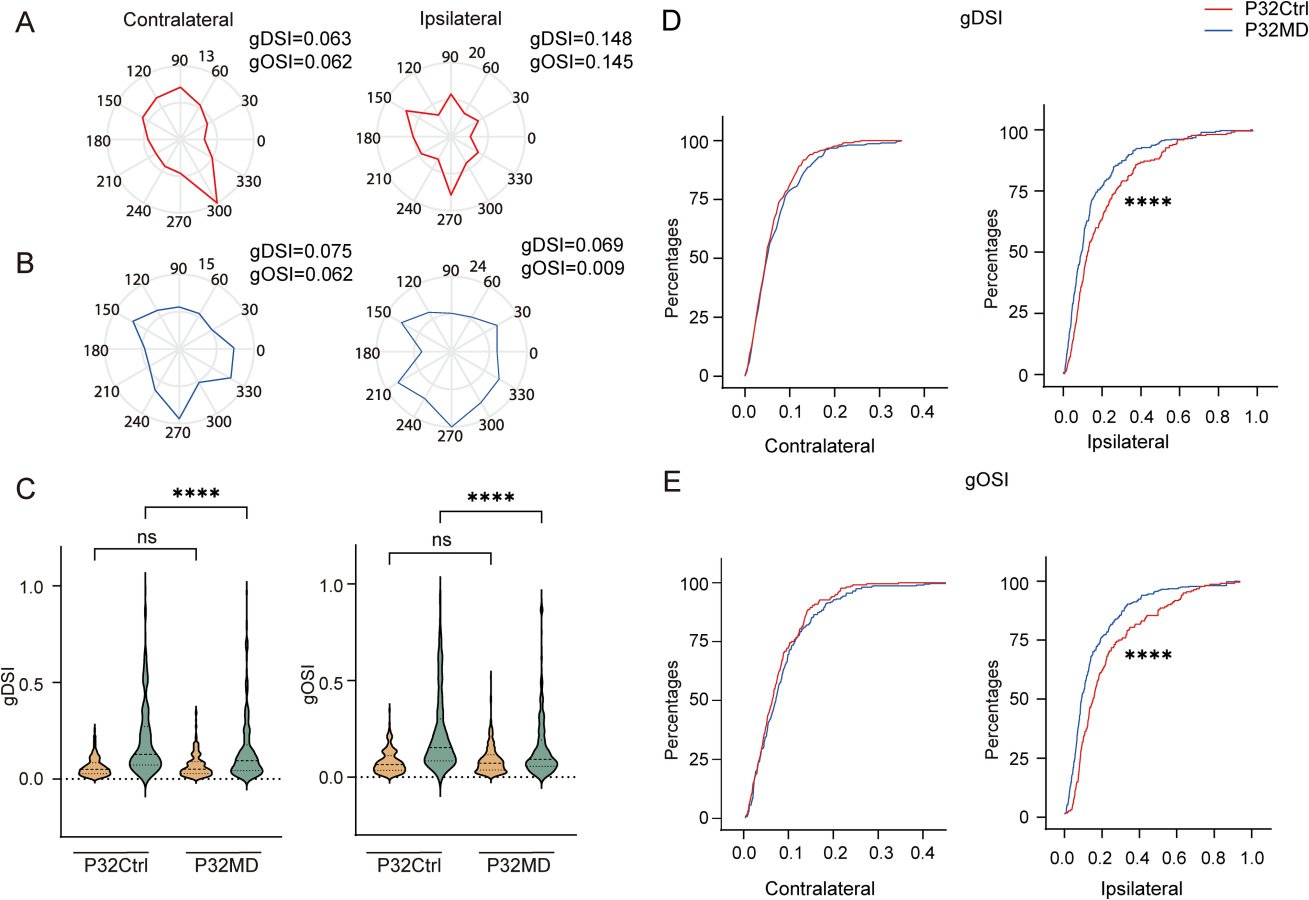
Four-day MD during the critical period reduced direction and orientation selectivity of ipsilateral eye responses in dLGN neurons. **(A,B)** Polar plots of example cells showing responses to contralateral (left) and ipsilateral (right) eye stimulation in control **(A)** and MD group **(B)**. **(C)** Comparisons of gDSI (left) and gOSI (right) of contralateral (yellow) and ipsilateral (green) eye responses in dLGN neurons between P32 Ctrl and P32 MD group, respectively. (Kruskal–Wallis test, P32 Ctrl, *n* = 220 cells from 10 mice; P32 MD, *n* = 318 cells from 10 mice). ^ns^*p* > 0.05 and ^****^p < 0.0001. **(D,E)** Comparisons of cumulative distribution of the gDSI (**D**, Ctrl vs. MD; contralateral eye: *p* = 0.5148, ipsilateral-eye: *p* = 0.0004) and gOSI (**E**, Ctrl vs. MD; contralateral-eye: *p* = 0.4474, ipsilateral-eye: *p* < 0.0001) of contralateral (left) and ipsilateral (right) eye responses between control (red) and MD group (blue) in P32. Kolmogorov–Smirnov test. ^ns^*p* > 0.05, ^***^p < 0.001, and ^****^p < 0.0001.

Neurons in the dLGN integrate inputs from retinal ganglion cells (RGCs), which encode distinct spatiotemporal features of the visual space, including direction selectivity and orientation selectivity (Kerschensteiner and Guido, 2017). Consequently, dLGN neurons inherit these functional properties, and relay orientation- and direction-tuned signals to specific cortical layers in V1 (Sun et al., 2016; Cruz-Martín et al., 2014). However, it remains unclear whether OD plasticity induced by MD during the critical period functionally modulates these receptive field properties and, if so, through what mechanisms. To address this, we next examined changes in DS and OS of dLGN neuronal responses to contralateral and ipsilateral eye stimulation following 4-day MD during the critical period (Figures 2A,B). We found that the direction and orientation selectivity of ipsilateral eye responses were significantly attenuated, while those of contralateral eye responses remained unaffected (Ctrl vs. MD for gDSI: contralateral eye: *p* = 0.7225, ipsilateral-eye: *p* < 0.0001; for gOSI: contralateral-eye: *p* = 0.4349, ipsilateral-eye: *p* < 0.0001; Figures 2C–E; Supplementary Figures S2A, S3A). Similarly, these impairments were consistently observed in both excitatory and inhibitory neuronal populations (Supplementary Figures S1F,G). Further, with increasing MD duration, the DS and OS of ipsilateral eye responses were continuously attenuated, and those of contralateral eye responses were significantly increased after 7-day MD (Supplementary Figures S2D,E).

Our results further demonstrated that the reduction in orientation and direction selectivity of ipsilateral eye responses in dLGN neurons following 4-day MD during the critical period was attributable to increased neuronal responses to ipsilateral stimulation across nearly all directions and orientations (Figures 2A,B). This suggests the excitability of the dLGN neurons to the ipsilateral eye stimulation increased, potentially resulting from down-regulation of intra-thalamic inhibitory signaling. Moreover, these changes in inhibitory circuits as a common regulatory mechanism causing elevated excitability to the ipsilateral eye stimulation were shared by both excitatory and inhibitory neurons. However, this proposed mechanism requires further experimental validation.

### 3.3 Down-regulation of intra-thalamic inhibitory components following 4-day MD during the critical period

**Figure 3:**
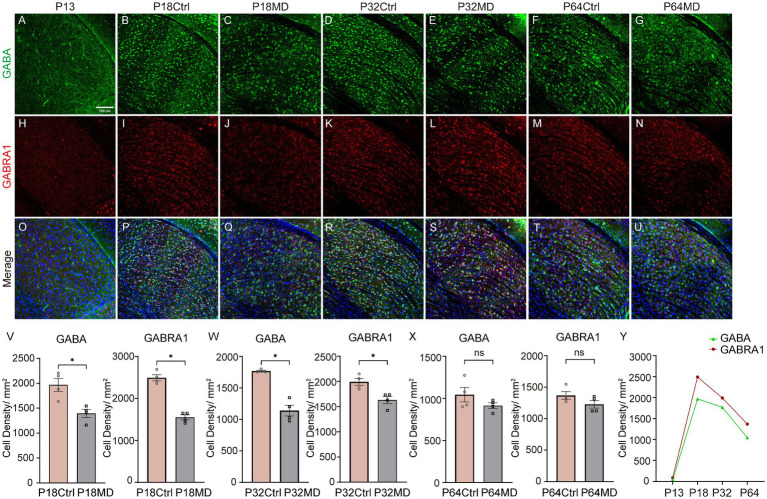
The developmental changes of densities of GABA^+^ neurons and GABRA1^+^ neurons and their down-regulation following 4-day MD during the critical period. **(A–U)** Expression of GABA **(A–G)**, GABRA1 **(H–N)**, and their merge **(O–U)** in dLGN at postnatal day (P)13, P18 Ctrl, P18 MD, P32 Ctrl, P32 MD, P64 Ctrl, and P64 MD. green, GABA; red, GABRA1; blue, DAPI. **(V–X)** The left plots are for GABA; the right plots are for GABRA1. **(V)** MD led to a decrease in the expression of GABA^+^ neurons and GABRA1^+^ neurons during the pre-CP (*n* = 4, respectively). **(W)** MD led to a decrease in the expression of GABA^+^ neurons and GABRA1^+^ neurons during the CP (*n* = 4, respectively). **(X)** MD did not alter the densities of GABA^+^ neurons and GABRA1^+^ neurons during the adulthood (*n* = 4, respectively). all unpaired *t*-test, two-tailed, respectively. Error bars represent mean ± SEM. ^ns^*p* > 0.05 and ^*^p < 0.05. **(Y)** Developmental changes in densities of GABA^+^ neurons and GABRA1^+^ neurons at different stages of mouse visual development. Scale bar, 100 μm.

Inhibitory neural circuits play a crucial role in the development of dLGN (Sommeijer et al., 2017). To investigate whether inhibitory components are associated with experience-dependent plasticity in dLGN, we next investigated the developmental changes of GABA positive and GABRA1 positive neuron densities and their expression changes following 4-day MD during different periods. The results revealed that both GABA^+^ and GABRA1^+^ neurons were scarce in P13 pups, but their expression levels rapidly increased with the onset of the critical period, and then declined with age after the peak of the critical period until adulthood (Figures 3A,B,D,F,H,I,K,M,O,P,R,T,Y). Furthermore, 4-day MD led to the expression decline of both GABA^+^ and GABRA1^+^ neurons during the critical period and pre-CP, while no changes in adulthood (Figures 3C,J,Q,E,L,S,G,N,U; Ctrl vs. MD: Pre-CP, GABA: 1967.0 ± 131.5 vs. 1395.0 ± 82.3, *p* = 0.0102, GABRA1: 2492.0 ± 74.4 vs. 1548.0 ± 60.2, *p* < 0.0001, Figure 3V; CP, GABA: 1768.0 ± 11.4 vs. 1139.0 ± 84.3, *p* = 0.0003, GABRA1: 1991.0 ± 69.0 vs. 1630.0 ± 72.7, *p* = 0.0113, Figure 3W; adulthood, GABA: 1044.0 ± 86.3 vs. 913.4 ± 34.1, *p* = 0.2095, GABRA1: 1369.0 ± 61.4 vs. 1227.0 ± 60.8, *p* = 0.1518, Figure 3X). The observed reductions in GABA^+^ and GABRA1^+^ neuron densities could result from either neuronal loss or downregulated GABA/GABRA1 synthesis. To distinguish these possibilities, we performed TUNEL staining on the dLGN and found that no apoptosis was detected in either the P32 Ctrl or P32 MD group (Supplementary Figures S3A–F). These findings imply that inhibitory circuits may play an important role in the regulation of experience-dependent plasticity of dLGN neurons, and that abnormal visual experience may affect the development of intra-thalamic inhibitory circuits.

### 3.4 Pharmacological activation of GABA_A_ receptors rescued the MD-induced OD shifts and feature selectivity impairments

**Figure 4:**
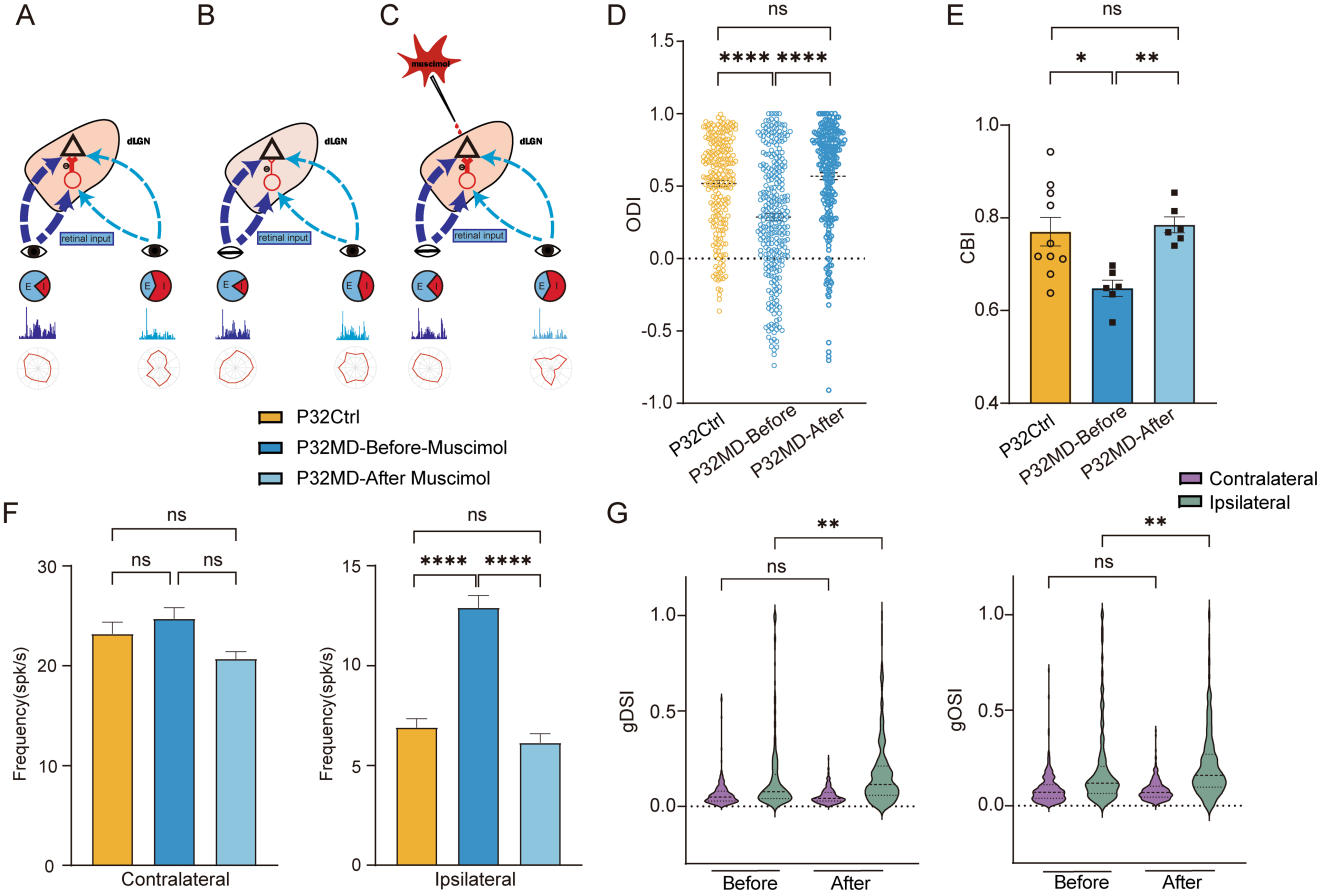
Schematic diagram for the hypothesis explaining how intra-thalamic GABAergic signaling mediates thalamic binocular plasticity during the critical period and pharmacological activation of GABA_A_ receptors rescued the MD-induced OD shifts and feature selectivity impairments. **(A)** The retina visual inputs from contralateral (dark blue) and ipsilateral (light blue) eye travel to the dLGN. dLGN neurons receive asymmetric excitatory inputs from the retinas of both eyes and intra-thalamic inhibitory synaptic inputs (red). **(B)** MD reduces inhibitory drive to dLGN neurons, disrupting excitation-inhibition (E/I) balance and elevating cellular excitability, which would disproportionately amplify responses of ipsilateral eye, thus leading to OD shift and reduction of feature selectivity of the ipsilateral eye responses. **(C)** Pharmacological activation of GABA_A_ receptors with muscimol restoring GABAergic inhibition could normalize E/I balance, thereby rescuing OD shifts and feature selectivity impairments. **(D)** Comparisons of ODI of dLGN neurons between P32 Ctrl, P32 MD before and after administration of muscimol (P32 Ctrl, *n* = 220 cells from 10 mice; P32 MD before muscimol, *n* = 283 cells from 6 mice; P32 MD after muscimol, *n* = 247 cells from 6 mice; respectively). **(E)** Comparisons of CBI between P32 Ctrl, P32 MD before and after administration of muscimol (P32 Ctrl vs. P32 MD before muscimol, *p* = 0.0124, unpaired *t*-test, two-tailed; P32 MD before muscimol vs. P32 MD after muscimol, *p* = 0.0013, paired *t*-test, two-tailed; P32 Ctrl vs. P32 MD after muscimol, *p* = 0.7302, unpaired *t*-test, two-tailed). **(F)** Comparisons of mean responses of contralateral (left) and ipsilateral eye (right) in dLGN neurons in P32 Ctrl and P32 MD mice before and after administration of muscimol. **(G)** Violin plot of gDSI (left) and gOSI (right) of contralateral (yellow) and ipsilateral (green) eye responses in dLGN neurons between before and after administration of muscimol. **(A–D)** Yellow represents P32 Ctrl, dark blue represents P32 MD before administration of muscimol, and light blue represents P32 MD after administration of muscimol. **(A,C–F)** Kruskal–Wallis test, respectively. ^ns^*p* > 0.05, ^*^p < 0.05, ^**^p < 0.01, and ^****^p < 0.0001. Error bars represent mean ± SEM.

To investigate whether reduced intra-thalamic GABAergic signaling mediates MD-induced OD shifts and degraded feature selectivity in dLGN neurons during the critical period, we pharmacologically enhanced GABA_A_ receptor activity. Our proposed mechanism posits that MD reduces inhibitory drive to dLGN neurons, disrupting excitation-inhibition (E/I) balance and elevating cellular excitability (Figures 4A,B). Given the retinogeniculate input asymmetry—where contralateral eye projections dominate over ipsilateral ones—we predicted that E/I imbalance would disproportionately amplify responses from the weaker ipsilateral pathway. This would manifest as a relative increase in ipsilateral eye-driven activity and degraded feature encoding (Figure 4B). We hypothesized that restoring GABAergic inhibition through activating postsynaptic GABA_A_ receptors could normalize E/I balance, thereby rescuing OD shifts and feature selectivity impairments (Figure 4C). To test this, we induced 4-day MD in P28 mice and performed *in vivo* electrophysiological recordings in the dLGN at P32 (P32 MD group). Following baseline measurements, we locally administered muscimol, a GABA_A_ receptor agonist, to enhance intra-thalamic inhibition while monitoring neuronal responses. The results indicated that muscimol treatment reversed OD shifts in P32 MD mice to control levels (ODI: Ctrl vs. MD-before-muscimol: *p* < 0.0001, MD-before-muscimol vs. MD-after-muscimol: *p* < 0.0001, Ctrl vs. MD-after-muscimol: *p* = 0.1111, Figure 4D; CBI: Ctrl vs. MD-before-muscimol: *p* = 0.0124, MD-before-muscimol vs. MD-after-muscimol: *p* = 0.0013, Ctrl vs. MD-after-muscimol: *p* = 0.7302, Figure 4E), eliminating the deprivation-induced bias toward the open eye. Ipsilateral eye-evoked responses, which were abnormally elevated post-MD, returned to baseline amplitudes (ipsilateral eye: Ctrl vs. MD-before-muscimol: *p* < 0.0001, MD-before-muscimol vs. MD-after-muscimol: *p* < 0.0001, Ctrl vs. MD-after-muscimol: *p* = 0.2885, Figure 4F). Direction and orientation selectivity for ipsilateral eye stimulation—previously impaired by MD—showed significant improvement post-treatment (MD-before-muscimol vs. MD-after-muscimol: gDSI, *p* = 0.0049, gOSI, *p* = 0.0011, Figure 4G). These results confirm that intra-thalamic GABAergic signaling critically regulates both OD plasticity and feature encoding in the dLGN during the critical period. The rescue of functional properties by muscimol supports the model where MD-induced E/I imbalance destabilizes thalamic processing, with targeted GABAergic activity restoration effectively counteracting these deficits.

### 3.5 Early compensatory inhibition from V1 feedback in pre-critical period mice maintained network stability

**Figure 5:**
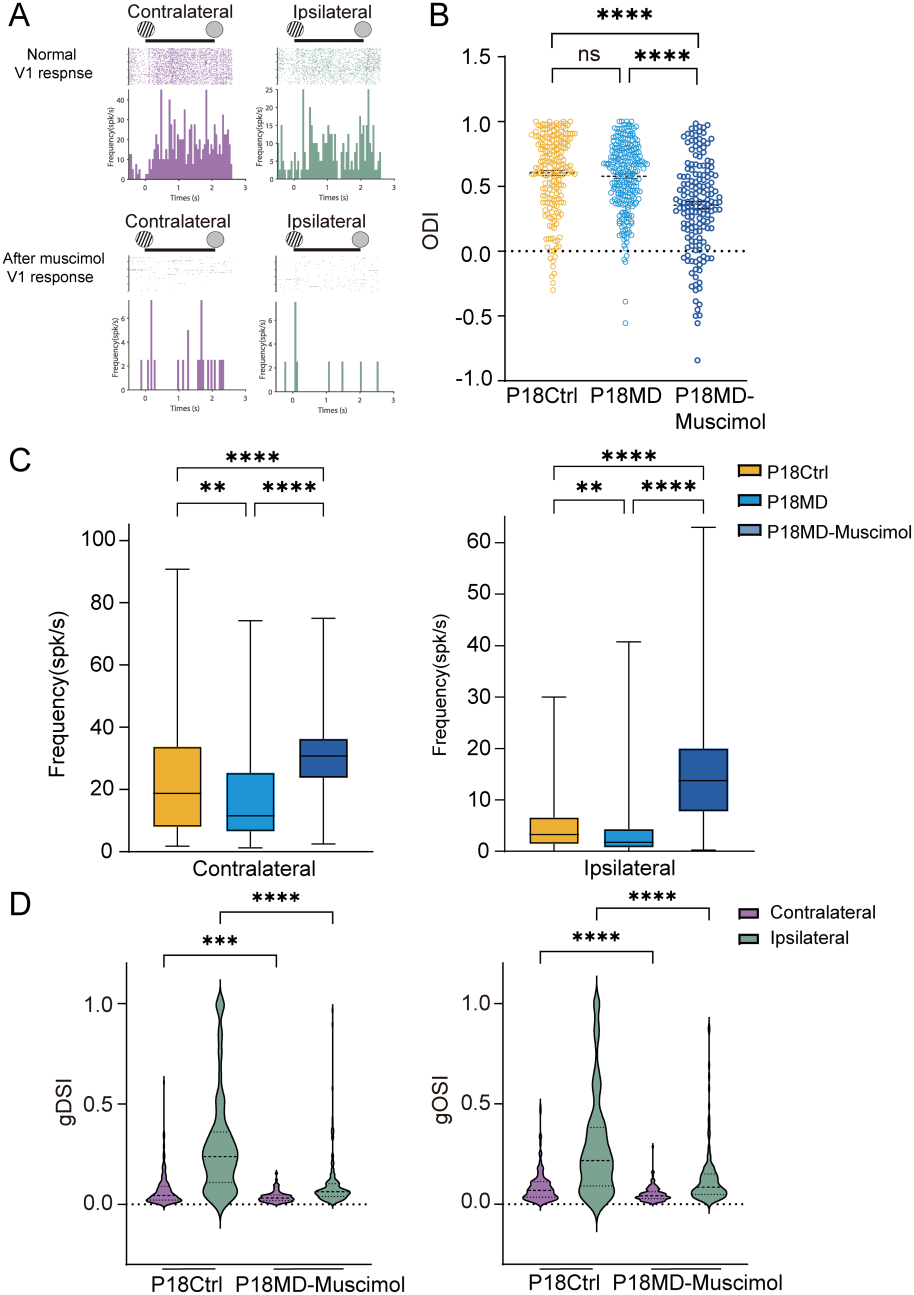
Early compensatory inhibition from V1 feedback in pre-critical period MD mice maintained network stability. **(A)** Examples of spike raster plots (for all directions) and peristimulus time histograms (PSTHs, for all direction) of a dLGN neuron before (top) and after (bottom) instant inactivation of V1 in response to full-field drifting grating to contralateral (left) and ipsilateral eye (right). Solid lines indicate 0–2 s after the onset of drifting grating stimulation. **(B)** Comparisons of ODI of dLGN neurons between P18 Ctrl, P18 MD and P18 MD with V1 instant inactivation of muscimol (P18 Ctrl, *n* = 203 cells from 7 mice; P18 MD, *n* = 238 cells from 8 mice; P18 MD with muscimol, *n* = 163 cells from 4 mice; respectively). **(C)** Comparisons of mean responses of contralateral (left) and ipsilateral eye (right) in dLGN neurons in P18 Ctrl (yellow), P18 MD (blue) and P18 MD with V1 instant inactivation of muscimol. **(D)** Violin plot of gDSI (left) and gOSI (right) of contralateral (purple) and ipsilateral (green) eye responses in dLGN neurons between P18 Ctrl and P18 MD with V1 instant inactivation of muscimol. **(B,C)** Yellow represents P18 Ctrl, blue represents P18 MD, and dark blue represents P18 MD with V1 instant inactivation of muscimol. **(B–D)** Kruskal–Wallis test; respectively. ^ns^*p* > 0.05, ^*^p < 0.05, ^**^p < 0.01, and ^****^p < 0.0001. Error bars represent mean ± SEM.

In the previous results, we observed the reduced GABA and GABRA1 expressions in P18 MD mice; however, the molecular alterations were not accompanied by corresponding OD changes (Figures 1E,F). This raises an important question about whether early inhibitory compensatory mechanisms mediated by V1 feedback may temporarily maintain network stability. To investigate whether V1 feedback to the dLGN compensates for reduced inhibitory signaling and maintains the neuronal activity levels in P18 MD mice, we performed instant V1 inactivation by applying muscimol (5 mM) to the V1 while recording dLGN responses (Figure 5A).

Our results reveal that instant V1 inactivation induced the significant OD shift toward the ipsilateral eye in P18 MD mice (Ctrl vs. MD for ODI: *p* = 0.5152, Ctrl vs. MD-muscimol: *p* < 0.0001; MD vs. MD-muscimol: *p* < 0.0001; Figure 5B), which was primarily due to a significantly stronger enhancement of the ipsilateral eye responses compared to the increase of contralateral eye responses (contralateral eye: Ctrl vs. MD-muscimol: *p* < 0.0001, MD vs. MD-muscimol: *p* < 0.0001; ipsilateral eye: Ctrl vs. MD-muscimol: *p* < 0.0001, MD vs. MD-muscimol: *p* < 0.0001; Figure 5C). These results indicate that the inhibitory input mediated by V1 feedback may take an important role in modulating the neuronal activity in the dLGN during early development. Besides, we found that 4-day MD in P18 mice did not induce the OD shift, but the responses to the contralateral eye and ipsilateral eye stimulation were both decreased (Ctrl vs. MD: contralateral eye: *p* = 0.0032, ipsilateral eye: *p* = 0.0015; Figure 5C), suggesting that MD during the pre-critical period induces a compensatory enhancement of V1 feedback-mediated inhibitory inputs to the dLGN.

Moreover, instant V1 inactivation also led to a marked decrease of direction and orientation selectivity of contralateral and ipsilateral eye responses in P18 MD mice (Ctrl vs. MD-muscimol: contralateral: gDSI, *p* = 0.0003, gOSI: *p* < 0.0001; ipsilateral: gDSI, *p* < 0.0001, gOSI, *p* < 0.0001; Figure 5D), which could be explained by the increase of overall neuronal excitability due to diminished V1-feedback mediated compensatory inhibition. This compensatory inhibition helps maintain the E/I balance and may mediates a partially homeostatic response in the developing dLGN in the pre-critical period, while V1-inactivation induced E/I imbalance would disproportionately amplify responses from the weaker ipsilateral pathway with OD shift toward the ipsilateral eye.

### 3.6 V1-feedback inactivation did not mask the rescue effect induced by pharmacological activation of intra-thalamic inhibitory circuits in critical period MD mice

**Figure 6:**
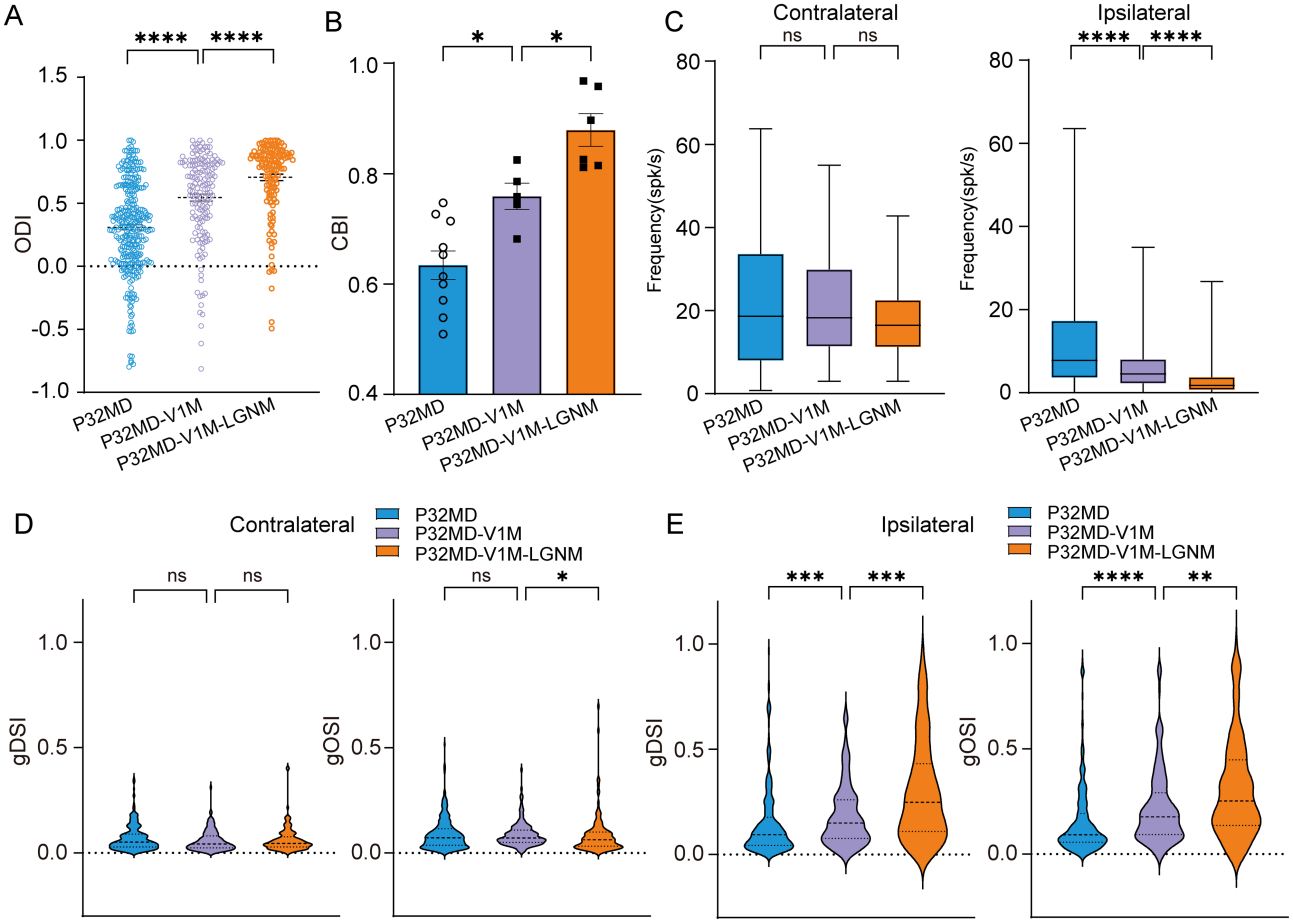
V1 feedback inactivation did not mask the rescue effect induced by pharmacological activation of dLGN GABA_A_ receptors in critical period MD mice. **(A)** Comparisons of ODI of dLGN neurons between P32 MD, P32 MD with instant inactivation of V1 using muscimol (5 mM) (P32MD-V1M), and P32 MD with V1 instant inactivation and activation of GABRA (P32MD-V1M-LGNM) (P32 MD, *n* = 318 cells from 10 mice; P32 MD-V1M, *n* = 175 cells from 5 mice; P32 MD-V1M-LGNM, *n* = 139 cells from 6 mice; respectively). **(B)** Comparisons of CBI of dLGN neurons between P32 MD, P32 MD-V1M and P32 MD-V1M-LGNM. **(C)** Comparisons of mean responses of contralateral (left) and ipsilateral eye (right) betweeen dLGN neurons in between P32 MD, P32 MD-V1M and P32 MD-V1M-LGNM. **(D–E)** Violin plot of gDSI (left) and gOSI (right) of contralateral **(D)** and ipsilateral **(E)** eye responses in dLGN neurons betweeen dLGN neurons in between P32 MD, P32 MD-V1M and P32 MD-V1M-LGNM. **(A–E)** Blue represents P32 MD, purple represents P32 MD-V1M, and orange represents P32 MD-V1M-LGNM. **(A,C,D)** Kruskal–Wallis test, respectively. **(B)** One way ANOVA test. ^ns^*p* > 0.05, ^*^p < 0.05, ^**^p < 0.01, and ^****^p < 0.0001. Error bars represent mean ± SEM.

One possibility of the reduction of inhibitory inputs in the dLGN neurons in critical period mice is the attenuation of the inputs from TRN which may be caused by decreased V1 excitatory inputs to TRN. At the same time, the V1 feedback provides direct excitatory input to the dLGN. Interestingly, we found that instant V1 inactivation using muscimol led to a recovery of OD shift toward the contralateral eye (MD vs. MD-V1-muscimol: for ODI: *p* < 0.0001, Figure 6A, for CBI: *p* = 0.0120, Figure 6B), with a decrease in the ipsilateral eye response in P32 MD mice (MD vs. MD-V1-muscimol: contralateral, *p* = 0.8234; ipsilateral, *p* < 0.0001, Figure 6C), which were consistent with a reported study (Qin et al., 2023). Besides, the DS and OS of ipsilateral eye responses were recovered with an overall decrease in responses (MD vs. MD-V1muscimol: gDSI, *p* = 0.0004; gOSI, *p* < 0.0001; Figure 6E). The reduction of ipsilateral eye responses after its inactivation cannot be attributed to the decrease of inhibition from V1 feedback, but rather result from loss of excitatory drive from V1. It suggests that V1 feedback to ipsilateral eye stimulation is enhanced in P32 MD mice, but the increase in excitation outweighs that of inhibition. This phenomenon requires further investigation in the future, for example, with *in vivo* whole-cell recording.

However, when combined with instant V1 inactivation, pharmacological activation of GABRA receptors in dLGN still maintained its effect in rescuing 4-day MD-induced OD shift and feature selectivity impairments in critical period mice (MD-V1-muscimol vs. MD-V1-muscimol-LGN-muscimol: ODI, < 0.0001, Figure 6A; CBI, *p* = 0.0286, Figure 6B; for contralateral: gDSI, *p* > 0.9999; gOSI, *p* = 0.0318; Figure 6D; for ipsilateral: gDSI, *p* = 0.0007; gOSI, *p* = 0.0067; Figure 6E) with more decrease of ipsilateral-eye responses (MD-V1-muscimol vs. MD-V1-muscimol-LGN-muscimol: contralateral, *p* = 0.1457; ipsilateral, *p* < 0.0001, Figure 6C). These results suggest that intra-thalamic inhibitory circuit mechanisms operate independently from V1 feedback in 4-day MD mice during the critical period.

### 3.7 Experience-dependent plasticity of receptive field properties in dLGN neurons was independent of variations in stimulus contrast and spatial frequency

Amblyopic eyes exhibit reduced contrast sensitivity, characterized by a decreased responsiveness to low-contrast stimuli, which is particularly pronounced at high spatial frequencies (Jia et al., 2024). Thus, the preserved responses to deprived-eye stimulation in dLGN neurons following 4-day MD during the critical period may be explained by the specific parametric selection of stimulus contrast and spatial frequency. To address this, we conducted recordings in both the control and 4-day MD groups at different developmental stages using stimuli with different contrast levels (1.0, 0.6, 0.3) and spatial frequencies (0.02, 0.05, 0.08 cycles/degree).

**Figure 7:**
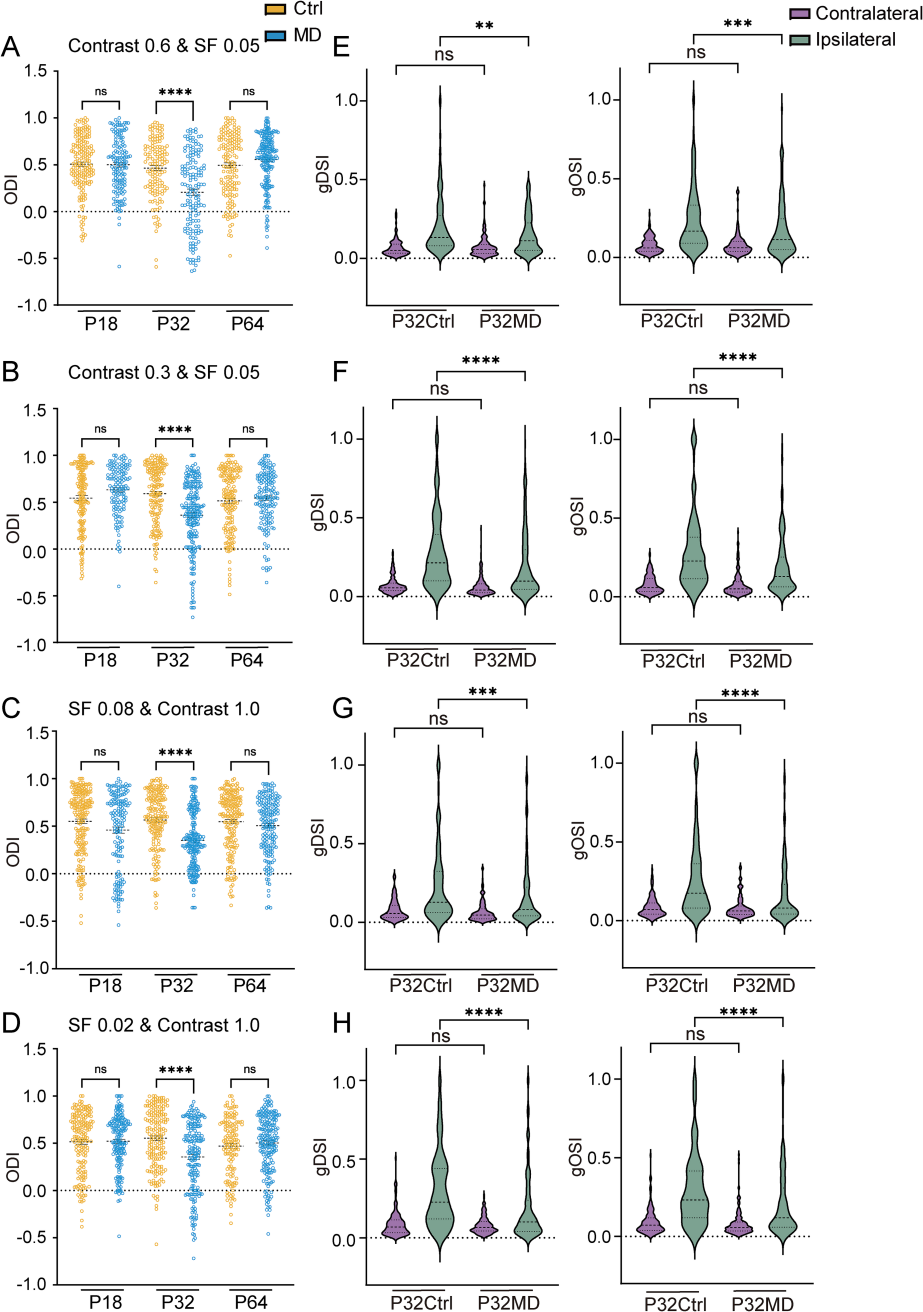
MD induced OD shifts and attenuations of DS and OS of ipsilateral eye responses in dLGN neurons were independent of variations in stimulus contrast and spatial frequency. **(A,B)** Comparisons of ODI in P18, P32, and P64 between control (yellow) and MD group (blue) with contrast of 0.6 **(A)** and 0.3 **(B)**. (Kruskal–Wallis test; for contrast 0.6, P18 Ctrl, *n* = 177 cells from 7 mice; P18 MD, *n* = 147 cells from 7 mice; P32 Ctrl, *n* = 141 cells from 7 mice; P32 MD, *n* = 152 cells from 7 mice; P64 Ctrl, *n* = 161 cells from 8 mice; P64 MD, *n* = 203 cells from 10 mice; for contrast 0.3, P18 Ctrl, *n* = 169 cells from 7 mice; P18 MD, *n* = 135 cells from 6 mice; P32 Ctrl, *n* = 164 cells from 8 mice; P32 MD, *n* = 211 cells from 8 mice; P64 Ctrl, *n* = 165 cells from 8 mice; P64 MD, *n* = 159 cells from 7 mice; respectively). ^ns^*p* > 0.05 and ^****^p < 0.0001. **(C,D)** Comparisons of ODI in P18, P32, and P64 between control (yellow) and MD group (blue) with SF 0.08 **(C)** and SF 0.02 **(D)** (Kruskal–Wallis test; for SF 0.08, P18 Ctrl, *n* = 195 cells from 8 mice; P18 MD, *n* = 154 cells from7 mice; P32 Ctrl, *n* = 177 cells from 8 mice; P32 MD, *n* = 214 cells from 7 mice; P64 Ctrl, *n* = 185 cells from 10 mice P64 MD, *n* = 180 cells from 9 mice; for SF 0.02, P18 Ctrl, *n* = 168 cells from 7 mice; P18 MD, *n* = 172 cells from 8 mice; P32 Ctrl, *n* = 176 cells from 8 mice; P32 MD, *n* = 189 cells from 7 mice; P64 Ctrl, *n* = 138 cells from 8 mice; P64 MD, *n* = 196 cells from 8 mice; respectively). ^ns^*p* > 0.05 and ^****^p < 0.0001. **(E–H)** The gDSI (left) and gOSI (right) of contralateral (purple) and ipsilateral (green) eye responses in dLGN neurons between P32 Ctrl and P32 MD in contrast 0.6 **(E)**, contrast 0.3 **(F)**, SF 0.08 **(G)**, SF 0.02 **(H)**. **(A–D)** Kruskal–Wallis test, respectively. ^ns^*p* > 0.05, ^**^p < 0.01, ^***^p < 0.001, and ^****^p < 0.0001.

Lastly, we observed that MD during the critical period induced OD shifts (Ctrl vs. MD for ODI: contrast 0.6, pre-CP: *p* > 0.9999, CP: *p* < 0.0001, adulthood: *p* > 0.2873, Figure 7A; contrast 0.3, pre-CP: *p* = 0.1534, CP: *p* < 0.0001, adulthood: *p* > 0.9999, Figure 7B; SF 0.08, pre-CP: *p* = 0.1208, CP: *p* < 0.0001, adulthood: *p* = 0.4202, Figure 7C; SF 0.02, pre-CP: *p* > 0.9999, CP: *p* < 0.0001, adulthood: *p* > 0.8666, Figure 7D; for CBI: Supplementary Figures S4C,D, S5C,D) and reduced DS and OS of ipsilateral eye responses (Ctrl vs. MD: contrast 0.6, gDSI: *p* = 0.0044, gOSI: *p* = 0.0005, Figure 7E; contrast 0.3, gDSI: *p* < 0.0001, gOSI: *p* < 0.0001, Figure 7F; SF 0.08, gDSI: *p* = 0.0004, gOSI: *p* < 0.0001, Figure 7G; SF 0.02: gDSI: *p* < 0.0001, gOSI: *p* < 0.0001, Figure 7H) across varying stimulus contrast and spatial frequency conditions. These experience-dependent alterations of receptive field properties were primarily driven by increased ipsilateral eye responses (Supplementary Figures S4E,F, S5E,F). However, a 4-day MD did not lead to OD shifts or changes in DS and OS of contralateral and ipsilateral eye responses during pre-CP and adulthood (Supplementary Figures S6A–E, S7A–E), except that at a high SF of 0.08 cycles/degree the DS and OS of ipsilateral eye responses decreased while no OD shift was observed after 4-day MD during pre-CP (Supplementary Figures S6A–E). In conclusion, the OD shift driven by ipsilateral response potentiation and the concomitant reduction in DS and OS of ipsilateral eye responses in dLGN neurons is independent of contrast and spatial frequency.

## 4 Discussion

In this study we identified experience-dependent plasticity of multiple receptive field properties in dLGN binocular neurons during the critical period, predominantly driven by alterations in ipsilateral eye responses, and proposed that such plasticity is mediated mainly through the modulation of intra-thalamic inhibitory circuits.

Object detection and feature processing are fundamental for mammals to navigate their environment efficiently and safely (Goodale and Milner, 1992). Visual information is encoded by neurons tuned to specific receptive field properties, including ocular dominance, orientation selectivity, direction selectivity, contrast sensitivity, and spatiotemporal tuning (Ringach et al., 2002). These properties serve as critical biomarkers for feature processing (Goetz et al., 2022; Tan et al., 2022; Krahe et al., 2011). Critical periods are essential for proper brain development and binocular visual functions (Hensch, 2005), and disruptions during these stages—such as maladaptive plasticity or suboptimal sensory inputs—can result in lifelong neurodevelopmental disorders, particularly those impairing binocular integration and depth perception (Hubel and Wiesel, 1970; Espinosa and Stryker, 2012; Tan et al., 2022).

The dLGN, a primary visual thalamic nucleus, is indispensable for feature perception. Emerging evidence have highlighted its capacity for binocular plasticity (Qin et al., 2023; Müllner and Roska, 2024). In line with recent works (Sommeijer et al., 2017; Li et al., 2023), we demonstrate that dLGN neurons are mostly binocular neurons and exhibit experience-dependent OD plasticity. A 4-day MD during the critical period induced a robust OD shift, challenging the classical view of the dLGN as a passive relay for retinal input to V1 with monocular neurons (Guzik-Kornacka et al., 2016; Sherman and Spear, 1982).

However, whether, when and how thalamic binocular plasticity induces changes in multiple receptive field properties and their underlying mechanisms remain unclear. Abnormal visual experience can lead to a series of functional changes in V1 (Pizzorusso et al., 2006; Cheng et al., 2022; Tan et al., 2022). It is well-known that OD plasticity in V1 occurs in two phases: an initial loss of deprived-eye (contralateral) responses followed by a gain in non-deprived-eye (ipsilateral) responses after prolonged deprivation (Frenkel and Bear, 2004). Furthermore, MD-induced declines in disparity selectivity in V1 are primarily observed in responses to the deprived eye (Scholl et al., 2017). Our study confirmed that a 4-day MD during the critical period can induce a robust OD shift in dLGN neurons, consistent with prior reports (Li et al., 2023; Sommeijer et al., 2017). However, this short-term MD induced OD shift arises from ipsilateral-eye response potentiation, independent of contrast and SF conditions, accompanied by reduced direction and orientation selectivity for the ipsilateral eye, with no change for the contralateral eye. These findings suggested that the plasticity mechanisms in dLGN may differ from those in V1.

Consistent with a previous study (Sommeijer et al., 2017), we showed that the OD shift toward the ipsilateral eye undergoes dynamic changes as MD duration increases. Interestingly, the deprived-eye responses increase but not decrease 2 days post-MD, suggesting that the reduction of inhibition within the dLGN occurs faster and earlier than the reduction in excitatory inputs to the dLGN, as the MD duration increases, the excitatory inputs in response to the contralateral eye stimulation begin to weaken, ultimately leading to a delayed emergence of decrease of the contralateral eye responses after 7 days of MD, which suggests that excitatory input from the deprived eye undergoes only weak or nonsignificant alterations during early MD. In contrast, the ipsilateral-eye responses of dLGN neurons showed a continuous increase starting at early MD with increasing MD duration. These dynamic changes reveal distinct developmental timelines of excitatory and inhibitory inputs converging on dLGN neurons.

Local inhibitory circuit in the dLGN plays a pivotal role in visual information processing and the shaping of receptive field properties (Wang et al., 2011; Su et al., 2020). Previous studies have shown that OD plasticity can be induced with long-term MD in the dLGN during the critical period and adulthood, which requires synaptic inhibition within the thalamus (Sommeijer et al., 2017; Qin et al., 2023). The GABA_A_ receptor α1 subunit plays a critical role in inhibitory circuits in both V1 and thalamus (Kralic et al., 2006; Sommeijer et al., 2017; Fagiolini et al., 2004). Consistent with prior research (Sommeijer et al., 2017), we found that GABRA1^+^ neurons were sparse at eye opening but rapidly increase in number with age, aligning with the rapid development of thalamic inhibition post-eye opening (Bickford et al., 2010). At the same time, we observed that the densities of both GABA^+^ neurons and GABRA1^+^ neurons peaked during the critical period, but were significantly reduced following 4-day MD. These findings highlight the essential role of GABA_A_ receptor-mediated inhibition in dLGN functional development.

To dissect the contribution of intra-thalamic inhibition to the observed alterations in receptive field properties in the dLGN, we pharmacologically enhanced GABAergic signaling. Notably, activating intra-thalamic inhibitory circuits via GABA_A_ receptor agonist restored the OD shifts and rescued direction/orientation selectivity deficits induced by MD during the critical period. Further, even after instant inactivation of V1, the rescue of MD-induced impairments via activating intra-thalamic inhibitory circuits still remained. These findings underscore the crucial role of thalamic inhibitory circuits in critical period plasticity.

However, these findings contrast with the observations in V1, where inhibitory circuit maturation drives the transition of OD plasticity from the critical period to adulthood, with heightened inhibition contributing to critical-period closure (van Versendaal and Levelt, 2016). The distinct role of dLGN inhibition suggests additional factors—such as corticothalamic inputs from V1 (Thompson et al., 2016; Liang and Chen, 2020)—may regulate critical-period onset/closure in the thalamus. The distinct plasticity mechanisms between the dLGN and V1 reveal the complexity of visual system development and cross-circuit interactions.

Additionally, besides the local intra-thalamic circuits, the V1 feedback also modulates the responses of dLGN neurons to contralateral or ipsilateral eye, which depends on whether excitatory or inhibitory projection dominates (Thompson et al., 2016; Denman and Contreras, 2015; Howarth et al., 2014; Olsen et al., 2012). Our results showed that the influence of V1 feedback on response properties is different at different developmental stages. Transient V1 inactivation in in pre-critical period MD mice induced an OD shift toward the ipsilateral eye, characterized by a stronger enhancement of ipsilateral eye responses relative to contralateral responses. In contrast, during the critical period, V1 inactivation-induced recovery of OD shifts in MD mice were associated with reduced ipsilateral eye responses. These phase-dependent alterations reflect a developmental transition in V1 feedback mechanisms: inhibitory compensation dominates in the pre-critical period, which helps maintain the stability of network activity in the dLGN, while strengthened excitatory drive overrides this inhibition during the critical period.

Our study has several limitations: (1) *in vivo* dLGN recordings required removal of overlying cortical and hippocampal tissue, potentially perturbing neural circuits; (2) electrophysiological heterogeneity between dLGN core and shell regions remains unaddressed and warrants future investigation; (3) whether changes in retinogeniculate inputs to dLGN neurons underlie the observed plasticity is unknown (Liang and Chen, 2020), although our demonstration of contralateral response attenuation in 7-day MD mice provides preliminary evidence for experience-dependent reorganization of excitatory drive from retina to dLGN.

In conclusion, we found that abnormal visual experience can cause a significant OD shift in binocular neurons in the dLGN. This OD plasticity is attributed to the potentiation of ipsilateral eye responses but not to the depression of deprived-eye responses, independent of contrast and SF conditions. Concurrently, the direction and orientation selectivity of ipsilateral eye responses in these neurons were dramatically reduced. Furthermore, GABAergic intra-thalamic circuits critically regulate this plasticity, emphasizing their role in shaping thalamic function during development. The compensatory inhibition from V1 feedback helps maintain the network stability with no OD changes in pre-critical period. Pharmacological activation of GABAA receptors rescued the MD-induced OD shifts and feature selectivity impairments in critical period MD mice, operating independently of the V1 feedback. These findings may enrich our understanding of experience-dependent thalamic binocular plasticity and its mechanistic divergence from cortical circuits, offering insights into visual system disorders involving thalamocortical dysregulation.

## Data Availability

The raw data supporting the conclusions of this article will be made available by the authors, without undue reservation.

## References

[ref1] Allen E. A. Freeman R. D. (2006). Dynamic spatial processing originates in early visual pathways. J. Neurosci. 26, 11763–11774. doi: 10.1523/jneurosci.3297-06.2006 17093097 PMC6674796

[ref2] Bauer J. Weiler S. Fernholz M. H. P. Laubender D. Scheuss V. Hübener M.. (2021). Limited functional convergence of eye-specific inputs in the retinogeniculate pathway of the mouse. Neuron 109, 2457–2468.e12. doi: 10.1016/j.neuron.2021.05.036 34146468

[ref3] Bickford M. E. Slusarczyk A. Dilger E. K. Krahe T. E. Kucuk C. Guido W. (2010). Synaptic development of the mouse dorsal lateral geniculate nucleus. J. Comp. Neurol. 518, 622–635. doi: 10.1002/cne.22223 20034053 PMC4278806

[ref4] Casagrande V. A. Boyd J. D. (1996). The neural architecture of binocular vision. Eye (Lond.) 10, 153–160. doi: 10.1038/eye.1996.40 8776442

[ref5] Cheng S. Butrus S. Tan L. Xu R. Sagireddy S. Trachtenberg J. T.. (2022). Vision-dependent specification of cell types and function in the developing cortex. Cell 185, 311–327.e24. doi: 10.1016/j.cell.2021.12.022 35063073 PMC8813006

[ref6] Cruz-Martín A. El-Danaf R. N. Osakada F. Sriram B. Dhande O. S. Nguyen P. L.. (2014). A dedicated circuit links direction-selective retinal ganglion cells to the primary visual cortex. Nature 507, 358–361. doi: 10.1038/nature12989 24572358 PMC4143386

[ref7] Denman D. J. Contreras D. (2015). Complex effects on *in vivo* visual responses by specific projections from mouse cortical layer 6 to dorsal lateral geniculate nucleus. J. Neurosci. 35, 9265–9280. doi: 10.1523/jneurosci.0027-15.2015 26109652 PMC4478248

[ref8] Douglas R. J. Martin K. A. (2004). Neuronal circuits of the neocortex. Annu. Rev. Neurosci. 27, 419–451. doi: 10.1146/annurev.neuro.27.070203.144152 15217339

[ref9] Espinosa J. S. Stryker M. P. (2012). Development and plasticity of the primary visual cortex. Neuron 75, 230–249. doi: 10.1016/j.neuron.2012.06.009 22841309 PMC3612584

[ref10] Fagiolini M. Fritschy J. M. Löw K. Möhler H. Rudolph U. Hensch T. K. (2004). Specific GABAA circuits for visual cortical plasticity. Science 303, 1681–1683. doi: 10.1126/science.1091032 15017002

[ref11] Frenkel M. Y. Bear M. F. (2004). How monocular deprivation shifts ocular dominance in visual cortex of young mice. Neuron 44, 917–923. doi: 10.1016/j.neuron.2004.12.003 15603735

[ref12] Gilbert C. D. Li W. Piech V. (2009). Perceptual learning and adult cortical plasticity. J. Physiol. 587, 2743–2751. doi: 10.1113/jphysiol.2009.171488 19525560 PMC2718234

[ref13] Gilbert C. D. Wiesel T. N. (1992). Receptive field dynamics in adult primary visual cortex. Nature 356, 150–152. doi: 10.1038/356150a0 1545866

[ref14] Goetz J. Jessen Z. F. Jacobi A. Mani A. Cooler S. Greer D.. (2022). Unified classification of mouse retinal ganglion cells using function, morphology, and gene expression. Cell Rep. 40:111040. doi: 10.1016/j.celrep.2022.111040 35830791 PMC9364428

[ref15] Goodale M. A. Milner A. D. (1992). Separate visual pathways for perception and action. Trends Neurosci. 15, 20–25. doi: 10.1016/0166-2236(92)90344-8 1374953

[ref16] Gordon J. A. Stryker M. P. (1996). Experience-dependent plasticity of binocular responses in the primary visual cortex of the mouse. J. Neurosci. 16, 3274–3286. doi: 10.1523/jneurosci.16-10-03274.1996 8627365 PMC6579137

[ref17] Guzik-Kornacka A. van der Bourg A. Vajda F. Joly S. Christ F. Schwab M. E.. (2016). Nogo-A deletion increases the plasticity of the optokinetic response and changes retinal projection organization in the adult mouse visual system. Brain Struct. Funct. 221, 317–329. doi: 10.1007/s00429-014-0909-3 25284126

[ref18] Hao X. Liu Q. Chan J. Li N. Shi X. Gu Y. (2022). Binocular visual experience drives the maturation of response variability and reliability in the visual cortex. iScience 25:104984. doi: 10.1016/j.isci.2022.104984 36105593 PMC9465340

[ref19] Hensch T. K. (2005). Critical period plasticity in local cortical circuits. Nat. Rev. Neurosci. 6, 877–888. doi: 10.1038/nrn1787 16261181

[ref20] Hensch T. K. Fagiolini M. (2005). Excitatory-inhibitory balance and critical period plasticity in developing visual cortex. Prog. Brain Res. 147, 115–124. doi: 10.1016/s0079-6123(04)47009-5 15581701

[ref21] Hooks B. M. Chen C. (2020). Circuitry underlying experience-dependent plasticity in the mouse visual system. Neuron 106, 21–36. doi: 10.1016/j.neuron.2020.01.031 32272065 PMC7251959

[ref22] Howarth M. Walmsley L. Brown T. M. (2014). Binocular integration in the mouse lateral geniculate nuclei. Curr. Biol. 24, 1241–1247. doi: 10.1016/j.cub.2014.04.014 24856206 PMC4046226

[ref23] Hu G. Chen A. Ye J. Liu Q. Wang J. Fan C.. (2024). A developmental critical period for ocular dominance plasticity of binocular neurons in mouse superior colliculus. Cell Rep. 43:113667. doi: 10.1016/j.celrep.2023.113667 38184852

[ref24] Hubel D. H. Wiesel T. N. (1962). Receptive fields, binocular interaction and functional architecture in the cat's visual cortex. J. Physiol. 160, 106–154. doi: 10.1113/jphysiol.1962.sp006837 14449617 PMC1359523

[ref25] Hubel D. H. Wiesel T. N. (1970). The period of susceptibility to the physiological effects of unilateral eye closure in kittens. J. Physiol. 206, 419–436. doi: 10.1113/jphysiol.1970.sp009022 5498493 PMC1348655

[ref26] Huh C. Y. L. Abdelaal K. Salinas K. J. Gu D. Zeitoun J. Figueroa Velez D. X.. (2020). Long-term monocular deprivation during juvenile critical period disrupts binocular integration in mouse visual thalamus. J. Neurosci. 40, 585–604. doi: 10.1523/jneurosci.1626-19.2019 31767678 PMC6961993

[ref27] Jaepel J. Hübener M. Bonhoeffer T. Rose T. (2017). Lateral geniculate neurons projecting to primary visual cortex show ocular dominance plasticity in adult mice. Nat. Neurosci. 20, 1708–1714. doi: 10.1038/s41593-017-0021-0 29184207

[ref28] Jia Y. Ye Q. Liu J. Feng L. Xu Z. He Y.. (2024). Associations between the cause of amblyopia and pre-treatment contrast sensitivity, stereoacuity, fixation, and nystagmus. Heliyon 10:e28857. doi: 10.1016/j.heliyon.2024.e28857 38596124 PMC11002286

[ref29] Kerschensteiner D. Guido W. (2017). Organization of the dorsal lateral geniculate nucleus in the mouse. Vis. Neurosci. 34:E008. doi: 10.1017/s0952523817000062 28965501 PMC6380502

[ref30] Kirchgessner M. A. Franklin A. D. Callaway E. M. (2020). Context-dependent and dynamic functional influence of corticothalamic pathways to first- and higher-order visual thalamus. Proc. Natl. Acad. Sci. U.S.A. 117, 13066–13077. doi: 10.1073/pnas.2002080117 32461374 PMC7293611

[ref31] Ko H. Cossell L. Baragli C. Antolik J. Clopath C. Hofer S. B.. (2013). The emergence of functional microcircuits in visual cortex. Nature 496, 96–100. doi: 10.1038/nature12015 23552948 PMC4843961

[ref32] Krahe T. E. El-Danaf R. N. Dilger E. K. Henderson S. C. Guido W. (2011). Morphologically distinct classes of relay cells exhibit regional preferences in the dorsal lateral geniculate nucleus of the mouse. J. Neurosci. 31, 17437–17448. doi: 10.1523/jneurosci.4370-11.2011 22131405 PMC6623799

[ref33] Kralic J. E. Sidler C. Parpan F. Homanics G. E. Morrow A. L. Fritschy J. M. (2006). Compensatory alteration of inhibitory synaptic circuits in cerebellum and thalamus of gamma-aminobutyric acid type A receptor alpha1 subunit knockout mice. J. Comp. Neurol. 495, 408–421. doi: 10.1002/cne.20866 16485284

[ref34] Li N. Gu Y. (2020). The visual pathway for binocular integration. Neurosci. Bull. 36, 1089–1091. doi: 10.1007/s12264-020-00506-6 32367252 PMC7475132

[ref35] Li N. Liu Q. Zhang Y. Yang Z. Shi X. Gu Y. (2023). Cortical feedback modulates distinct critical period development in mouse visual thalamus. iScience 26:105752. doi: 10.1016/j.isci.2022.105752 36590174 PMC9794980

[ref36] Liang L. Chen C. (2020). Organization, function, and development of the mouse retinogeniculate synapse. Annu. Rev. Vis. Sci. 6, 261–285. doi: 10.1146/annurev-vision-121219-081753 32936733

[ref37] Litvina E. Y. Chen C. (2017). Functional convergence at the retinogeniculate synapse. Neuron 96, 330–338.e5. doi: 10.1016/j.neuron.2017.09.037 29024658 PMC5726778

[ref38] Livingstone M. Hubel D. (1988). Segregation of form, color, movement, and depth: anatomy, physiology, and perception. Science 240, 740–749. doi: 10.1126/science.3283936 3283936

[ref39] Müllner F. E. Roska B. (2024). Individual thalamic inhibitory interneurons are functionally specialized toward distinct visual features. Neuron 112, 2765–2782.e9. doi: 10.1016/j.neuron.2024.06.001 38917805 PMC11348917

[ref40] Olsen S. R. Bortone D. S. Adesnik H. Scanziani M. (2012). Gain control by layer six in cortical circuits of vision. Nature 483, 47–52. doi: 10.1038/nature10835 22367547 PMC3636977

[ref41] Piscopo D. M. El-Danaf R. N. Huberman A. D. Niell C. M. (2013). Diverse visual features encoded in mouse lateral geniculate nucleus. J. Neurosci. 33, 4642–4656. doi: 10.1523/jneurosci.5187-12.2013 23486939 PMC3665609

[ref42] Pizzorusso T. Medini P. Landi S. Baldini S. Berardi N. Maffei L. (2006). Structural and functional recovery from early monocular deprivation in adult rats. Proc. Natl. Acad. Sci. U.S.A. 103, 8517–8522. doi: 10.1073/pnas.0602657103 16709670 PMC1482523

[ref43] Priebe N. J. Ferster D. (2012). Mechanisms of neuronal computation in mammalian visual cortex. Neuron 75, 194–208. doi: 10.1016/j.neuron.2012.06.011 22841306 PMC3477598

[ref44] Qin Y. Ahmadlou M. Suhai S. Neering P. de Kraker L. Heimel J. A.. (2023). Thalamic regulation of ocular dominance plasticity in adult visual cortex. eLife 12:RP88124. doi: 10.7554/eLife.88124 37796249 PMC10554735

[ref45] Ringach D. L. Shapley R. M. Hawken M. J. (2002). Orientation selectivity in macaque V1: diversity and laminar dependence. J. Neurosci. 22, 5639–5651. doi: 10.1523/jneurosci.22-13-05639.2002 12097515 PMC6758222

[ref46] Rompani S. B. Müllner F. E. Wanner A. Zhang C. Roth C. N. Yonehara K.. (2017). Different modes of visual integration in the lateral geniculate nucleus revealed by single-cell-initiated transsynaptic tracing. Neuron 93, 767–776.e6. doi: 10.1016/j.neuron.2017.01.028 28231464 PMC5330803

[ref47] Rose T. Bonhoeffer T. (2018). Experience-dependent plasticity in the lateral geniculate nucleus. Curr. Opin. Neurobiol. 53, 22–28. doi: 10.1016/j.conb.2018.04.016 29733916

[ref48] Scholl B. Pattadkal J. J. Priebe N. J. (2017). Binocular disparity selectivity weakened after monocular deprivation in mouse V1. J. Neurosci. 37, 6517–6526. doi: 10.1523/jneurosci.1193-16.2017 28576937 PMC5511882

[ref49] Sherman S. M. Spear P. D. (1982). Organization of visual pathways in normal and visually deprived cats. Physiol. Rev. 62, 738–855. doi: 10.1152/physrev.1982.62.2.738 6280221

[ref50] Shi X. Barchini J. Ledesma H. A. Koren D. Jin Y. Liu X.. (2017). Retinal origin of direction selectivity in the superior colliculus. Nat. Neurosci. 20, 550–558. doi: 10.1038/nn.4498 28192394 PMC5374021

[ref51] Shi X. Jin Y. Cang J. (2018). Transformation of feature selectivity from membrane potential to spikes in the mouse superior colliculus. Front. Cell. Neurosci. 12:163. doi: 10.3389/fncel.2018.00163 29970991 PMC6018398

[ref52] Sommeijer J. P. Ahmadlou M. Saiepour M. H. Seignette K. Min R. Heimel J. A.. (2017). Thalamic inhibition regulates critical-period plasticity in visual cortex and thalamus. Nat. Neurosci. 20, 1715–1721. doi: 10.1038/s41593-017-0002-3 29184199

[ref53] Su J. Charalambakis N. E. Sabbagh U. Somaiya R. D. Monavarfeshani A. Guido W.. (2020). Retinal inputs signal astrocytes to recruit interneurons into visual thalamus. Proc. Natl. Acad. Sci. U.S.A. 117, 2671–2682. doi: 10.1073/pnas.1913053117 31964831 PMC7007527

[ref54] Sun W. Tan Z. Mensh B. D. Ji N. (2016). Thalamus provides layer 4 of primary visual cortex with orientation- and direction-tuned inputs. Nat. Neurosci. 19, 308–315. doi: 10.1038/nn.4196 26691829 PMC4731241

[ref55] Tan L. Ringach D. L. Trachtenberg J. T. (2022). The development of receptive field tuning properties in mouse binocular primary visual cortex. J. Neurosci. 42, 3546–3556. doi: 10.1523/jneurosci.1702-21.2022 35296547 PMC9053846

[ref56] Thompson A. D. Picard N. Min L. Fagiolini M. Chen C. (2016). Cortical feedback regulates feedforward retinogeniculate refinement. Neuron 91, 1021–1033. doi: 10.1016/j.neuron.2016.07.040 27545712 PMC5156570

[ref57] van Versendaal D. Levelt C. N. (2016). Inhibitory interneurons in visual cortical plasticity. Cell. Mol. Life Sci. 73, 3677–3691. doi: 10.1007/s00018-016-2264-4 27193323 PMC5002041

[ref58] Vaney D. I. Sivyer B. Taylor W. R. (2012). Direction selectivity in the retina: symmetry and asymmetry in structure and function. Nat. Rev. Neurosci. 13, 194–208. doi: 10.1038/nrn3165 22314444

[ref59] Voss P. (2013). Sensitive and critical periods in visual sensory deprivation. Front. Psychol. 4:664. doi: 10.3389/fpsyg.2013.00664 24133469 PMC3783842

[ref60] Wang X. Sommer F. T. Hirsch J. A. (2011). Inhibitory circuits for visual processing in thalamus. Curr. Opin. Neurobiol. 21, 726–733. doi: 10.1016/j.conb.2011.06.004 21752634 PMC3767471

[ref61] Zeater N. Cheong S. K. Solomon S. G. Dreher B. Martin P. R. (2015). Binocular visual responses in the primate lateral geniculate nucleus. Curr. Biol. 25, 3190–3195. doi: 10.1016/j.cub.2015.10.033 26778654

