# Supplementary material for: Experience-dependent plasticity of multiple receptive field properties in lateral geniculate binocular neurons during the critical period
Source: Front Cell Neurosci. 2025 Apr 28;19:1574505. doi: 10.3389/fncel.2025.1574505 (PMC12066550; doi:10.3389/fncel.2025.1574505)
Supplement: Supplementary file 1 [file Data_Sheet_1.docx]

**Supplementary file**

**Experience-dependent plasticity of multiple receptive field properties in lateral geniculate binocular neurons during the critical period**

Meng Pan^1,*^, Jingjing Ye^1,*^, Yijing Yan^1,*^, Ailin Chen^1^, Xinyu Li^1^, Xin Jiang^1,3^, Wei Wang^1^, Xin Meng^1^, Shujian Chen^1^, Yu Gu^2, †^, Xuefeng Shi^1,3, †^

^1^Tianjin Key Laboratory of Ophthalmology and Visual Science, Tianjin Eye Institute, Tianjin Eye Hospital, Clinical College of Ophthalmology, Tianjin Medical University, Tianjin 300020, China.

^2^State Key Laboratory of Medical Neurobiology and MOE Frontiers Center for Brain Science, Institutes of Brain Science, Fudan University, Shanghai 200032, China.

^3^School of Medicine, Nankai University, Tianjin 300071, China.

^*^ These authors contributed equally to this work.

^†^ Corresponding authors

**Correspondence:**

Yu Gu, State Key Laboratory of Medical Neurobiology and MOE Frontiers Center for Brain Science, Institutes of Brain Science, Fudan University, No. 131. Dongan Road,

Xuhui District, Shanghai 200032, P.R. China;

Email: guyu_@fudan.edu.cn.

Xuefeng Shi, Tianjin Medial University, Tianjin Eye Hospital, No. 4 Gansu Road, Heping District, Tianjin 300020, P.R. China.

Email: shixf_tmu@163.com

**
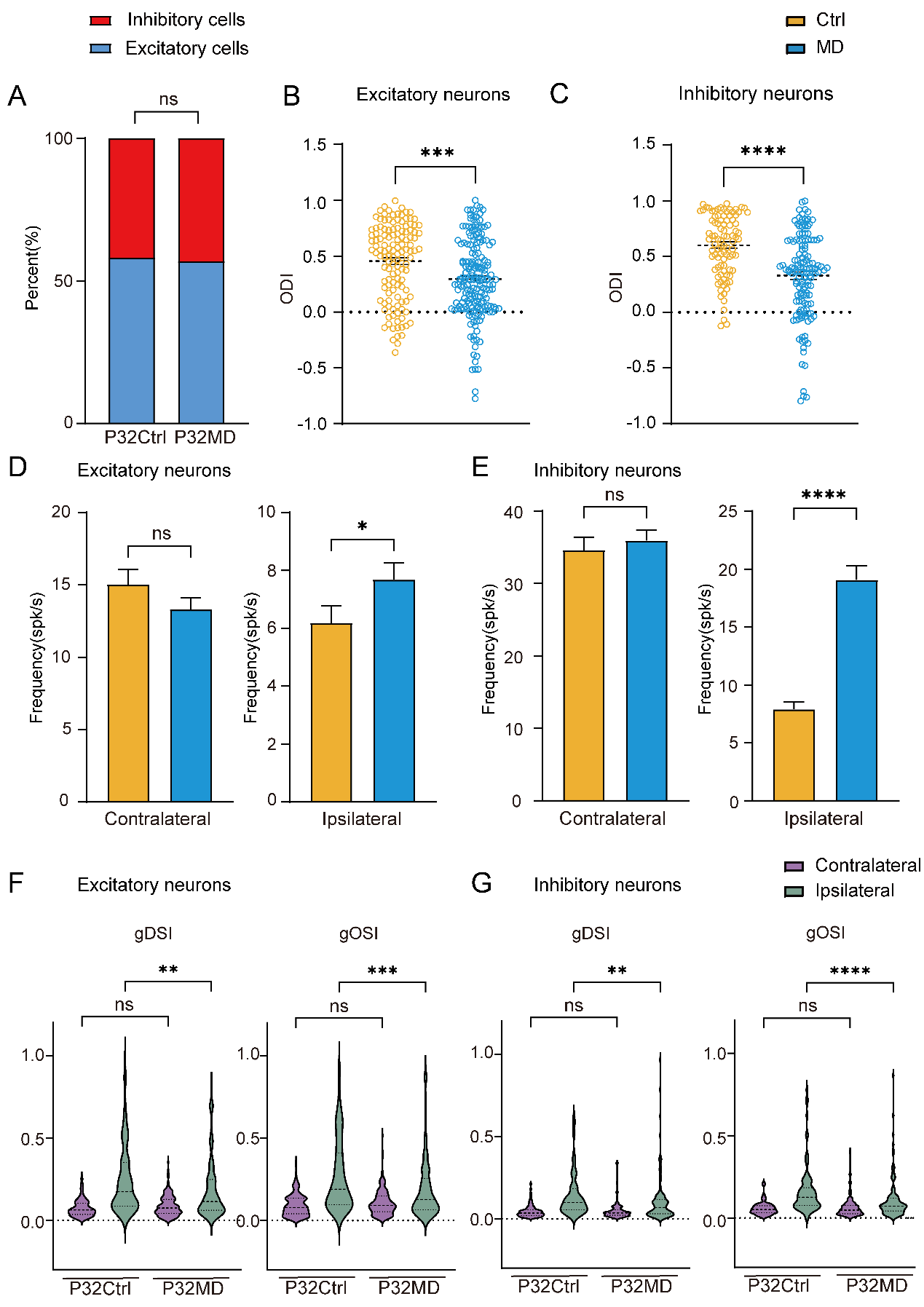
**

**Figure S1 Both excitatory and inhibitory neurons exhibited OD shifts to ipsilateral eye, and reduction of direction and orientation selectivity of ipsilateral eye responses subsequent to 4-day MD during the critical period.**

**A** The mean percentage of the excitatory (blue) and inhibitory (red) neurons was unchanged after MD (*P* = 0.7707, Chi-square test).

**B and C** Comparisons of ODI in excitatory (B, *P* = 0.0001; P32 Ctrl, *n* = 128 cells from 10 mice; P32 MD, *n* = 181 cells from 10 mice) and inhibitory (C, *P* < 0.0001; P32 Ctrl, *n* = 92 cells from 10 mice; P32 MD, *n* = 137 cells from 10 mice) dLGN neurons between control (yellow) and MD group (blue).

**D and E** Comparisons of mean response in excitatory (D; contralateral, *P* = 0.1301; ipsilateral, *P*=0.0231) and inhibitory (E; contralateral, *P* = 0.5233; ipsilateral, *P* < 0.0001) dLGN neurons between P32 Ctrl and P32 MD mice for contralateral (left) and ipsilateral eye (right).

**F** Comparisons of gDSI (left) and gOSI (right) of contralateral (purple) and ipsilateral (green) eye responses in excitatory dLGN neurons between control and MD group (P32 Ctrl vs. P32 MD for gDSI, contralateral, *P* = 0.2458; ipsilateral, *P* = 0.0020; for gOSI, contralateral, *P* = 0.3006; ipsilateral, *P* = 0.0003; respectively).

**G** Comparisons of gDSI (left) and gOSI (right) of contralateral and ipsilateral eye responses in inhibitory dLGN neurons between control and MD group (P32 Ctrl vs. P32 MD for gDSI, contralateral, *P* > 0.9999; ipsilateral, *P* = 0.0011; for gOSI, contralateral, *P* > 0.9999; ipsilateral, *P* < 0.0001; respectively). Error bars represent mean ± SEM. B-E, Mann-Whitney U test; F and G, Kruskal-Wallis test, respectively. ns *P*>0.05, **P*<0.05, ** *P*<0.01 and *** *P*<0.001, **** *P*<0.0001, respectively.
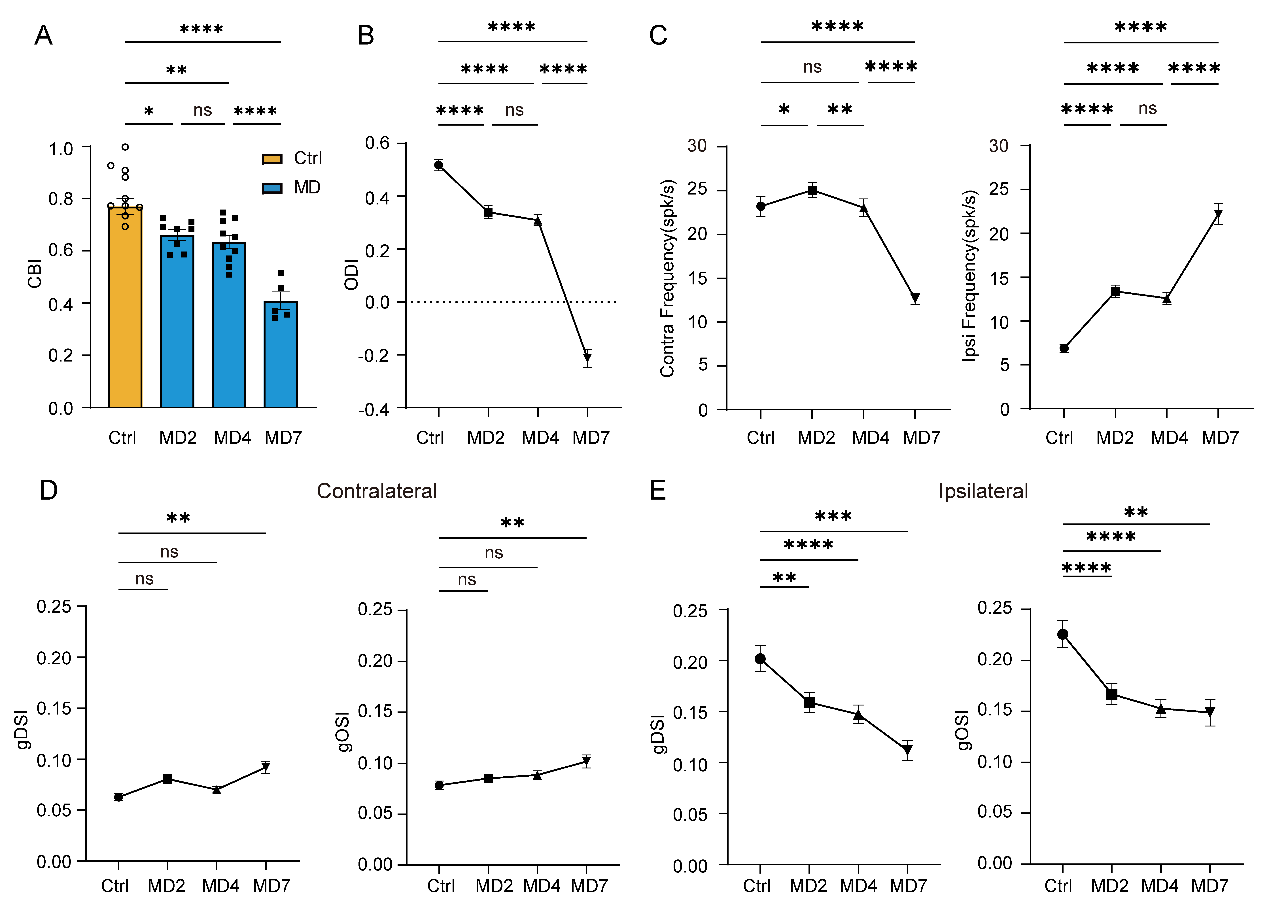


**Figure S2. The dynamic changes of OD shift, the response and direction/orientation selectivity for contralateral and ipsilateral eye with the MD durations.**

**A** Comparisons of CBI in critical period between Ctrl, 2-day MD, 4day-MD and 7-day MD. (Ctrl, *n*=220 cells from 10 mice; 2-day MD, *n*=256 cells from 8 mice; 4-day MD, *n*=318 cells from 10 mice;7-day MD, *n*=107 cells from 5 mice; respectively) (One way ANOVA test; Ctrl vs. 2MD, *P* = 0.0402; Ctrl vs. 4MD, *P* = 0.0038; Ctrl vs. 7MD, *P* < 0.0001; 2MD vs. 4MD, *P* = 0.9640; 4MD vs. 7MD, *P* < 0.0001).

**B** Comparisons of ODI in critical period between Ctrl, 2-day MD, 4day-MD and 7-day MD. (Kruskal-Wallis test; Ctrl vs. 2MD, *P* < 0.0001; Ctrl vs. 4MD, *P* < 0.0001; Ctrl vs. 7MD, *P* < 0.0001; 2MD vs. 4MD, *P* > 0.9999; 4MD vs. 7MD, *P* < 0.0001).

**C** Comparisons of mean responses of contralateral (left) and ipsilateral eye (right) in dLGN neurons between Ctrl, 2-day MD, 4day-MD and 7-day MD. (contralateral: Ctrl vs. 2MD, *P* = 0.0215; Ctrl vs. 4MD, *P* > 0.9999; Ctrl vs. 7MD, *P* < 0.0001; 2MD vs. 4MD, *P* = 0.0017; 4MD vs. 7MD, *P* < 0.0001; ipsilateral: Ctrl vs. 2MD, *P* < 0.0001; Ctrl vs. 4MD, *P* < 0.0001; Ctrl vs. 7MD, *P* < 0.0001; 2MD vs. 4MD, *P* = 0.6819; 4MD vs. 7MD, *P* < 0.0001;respectively).

**D** and **E** Comparisons of gDSI (left) and gOSI (right) of contralateral (D) and ipsilateral (E) eye responses in dLGN neurons between Ctrl, 2-day MD, 4day-MD and 7-day MD (contralateral for gDSI: Ctrl vs. 2MD, *P* = 0.0593; Ctrl vs. 4MD, *P* > 0.9999; Ctrl vs. 7MD, *P* < 0.0001; for gOSI: Ctrl vs. 2MD, *P* > 0.9999; Ctrl vs. 4MD, *P* = 0.5414; Ctrl vs. 7MD, *P* = 0.0015; ipsilateral for gDSI: Ctrl vs. 2MD, *P* = 0.0025; Ctrl vs. 4MD, *P* < 0.0001; Ctrl vs. 7MD, *P* = 0.0001; for gOSI: Ctrl vs. 2MD, *P* < 0.0001; Ctrl vs. 4MD, *P* < 0.0001; Ctrl vs. 7MD, *P* < 0.0084; respectively). Error bars represent mean ± SEM. B-E, Kruskal-Wallis test, respectively. ns *P*>0.05, **P*<0.05, ** *P*<0.01 and *** *P*<0.001, **** *P*<0.0001, respectively.

**
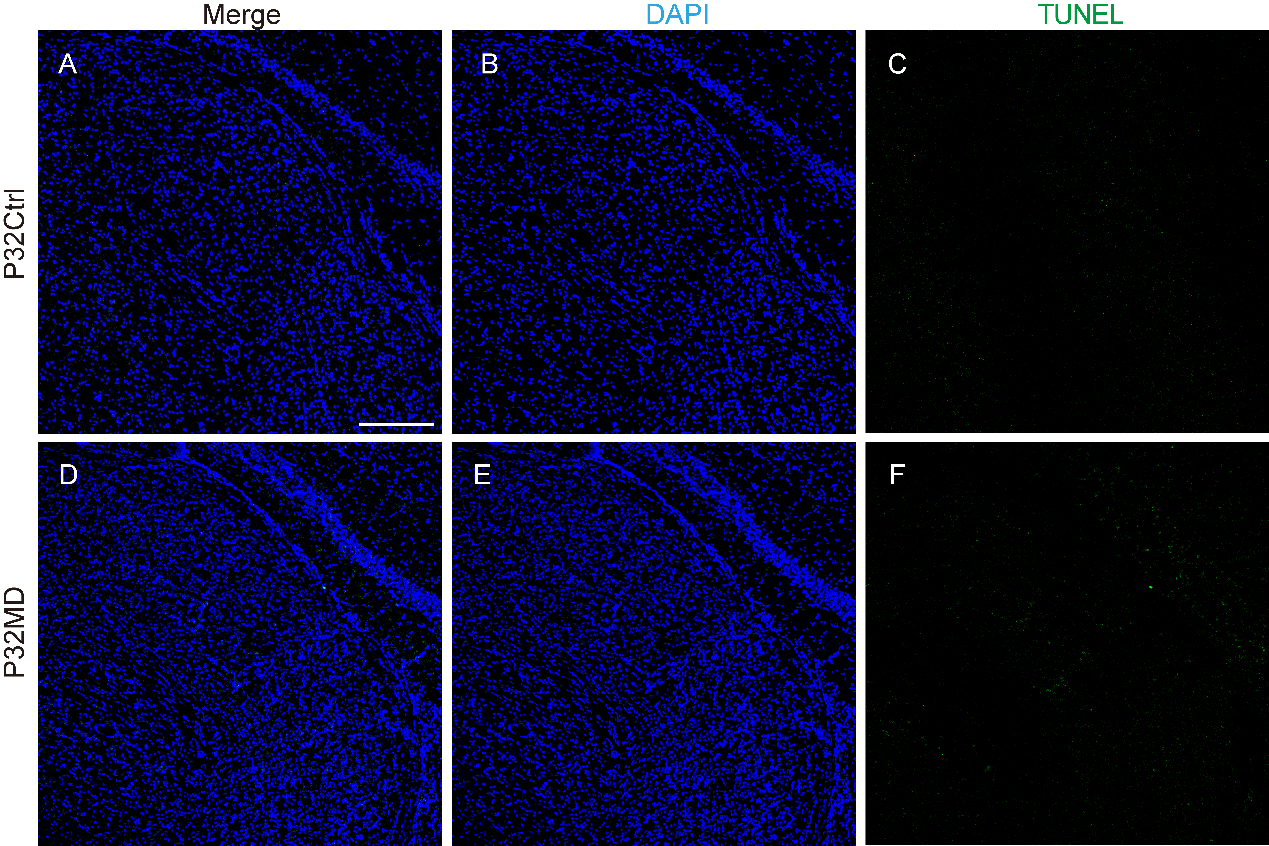
**

**Figure S3. The TUNEL staining in Ctrl and MD mice during the critical period.**

**A-F** DAPI (B, E), TUNEL (C, F), and their merge (A, D) in dLGN at P32Ctrl (top) and P32MD (bottom). blue, DAPI; green, TUNEL. Scale bar, 200 μm.

**
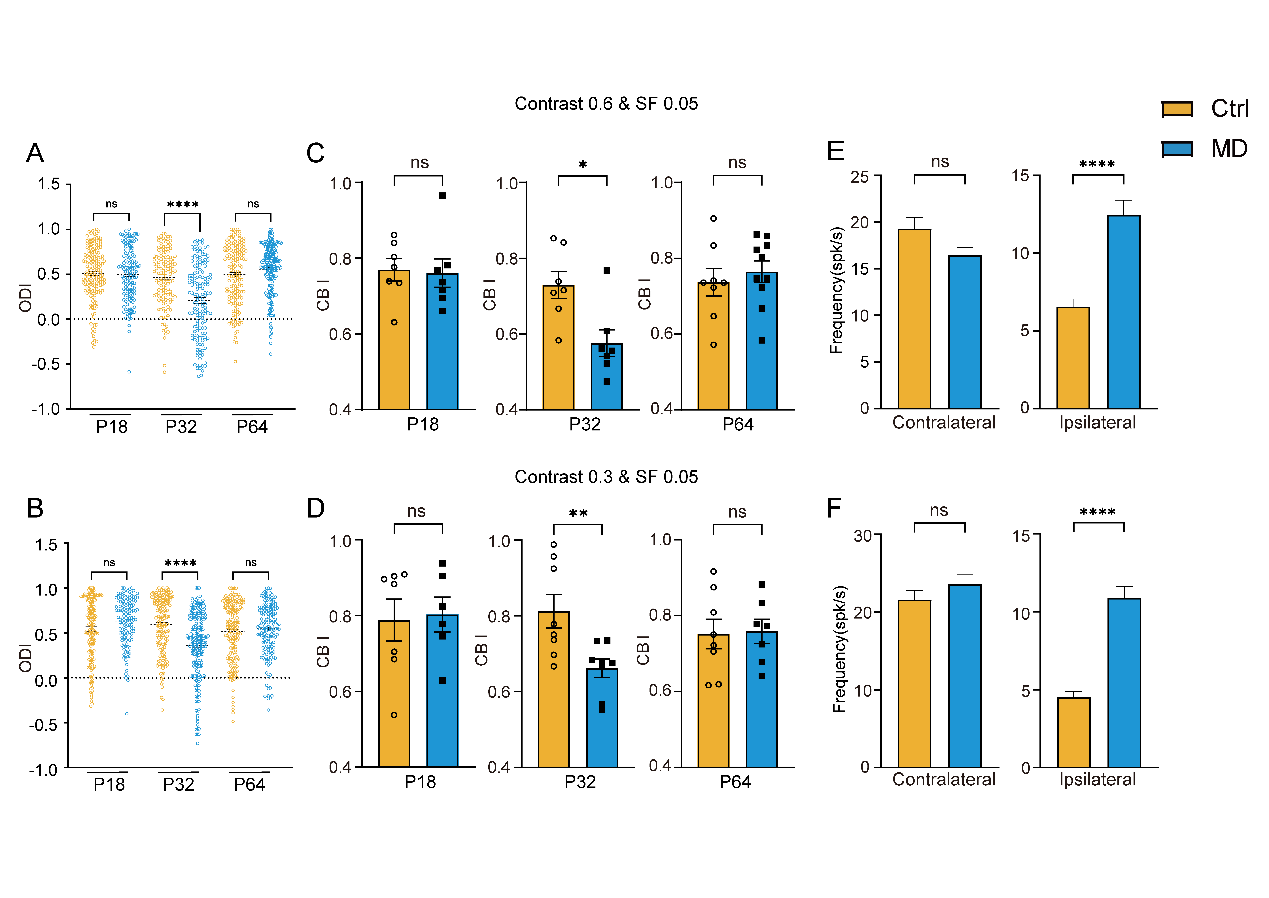
**

**Figure S4. MD induced OD shifts in dLGN neurons were independent of stimulus contrast levels.**

**A and B** Comparisons of ODI in P18, P32, and P64 between control (yellow) and MD group (blue) with contrast of 0.6 (A) and 0.3 (B). (Kruskal-Wallis test; for contrast 0.6, P18 Ctrl, *n*=177 cells from 7 mice; P18 MD, *n*=147 cells from 7 mice; P32 Ctrl, *n*=141 cells from 7 mice; P32 MD, *n*=152 cells from 7 mice; P64 Ctrl, *n*=161 cells from 8 mice; P64 MD, *n*=203 cells from 10 mice; for contrast 0.3, P18 Ctrl, *n*=169 cells from 7 mice; P18 MD, *n*=135 cells from 6 mice; P32 Ctrl, *n*=164 cells from 8 mice; P32 MD, *n*=211 cells from 8 mice; P64 Ctrl, *n*=165 cells from 8 mice; P64 MD, *n*=159 cells from 7 mice; respectively). ns: *P* > 0.05, *****P* < 0.0001.

**C and D** Comparisons of CBI between control and group for P18 (left), P32 (middle), and P64 (right) in contrast 0.6 (C) and in contrast 0.3 (D). Unpaired *t* test, two-tailed.ns *P* > 0.05, **P* <0.05, ***P* < 0.01.

**E and F** Comparisons of mean responses of contralateral (left) and ipsilateral eye (right) in dLGN neurons of P32 Ctrl and P32 MD mice in contrast 0.6 (E) and in contrast 0.3 (F). Mann-Whitney *U* test. ns *P* > 0.05, *****P* < 0.0001. Error bars represent mean ± SEM.

**
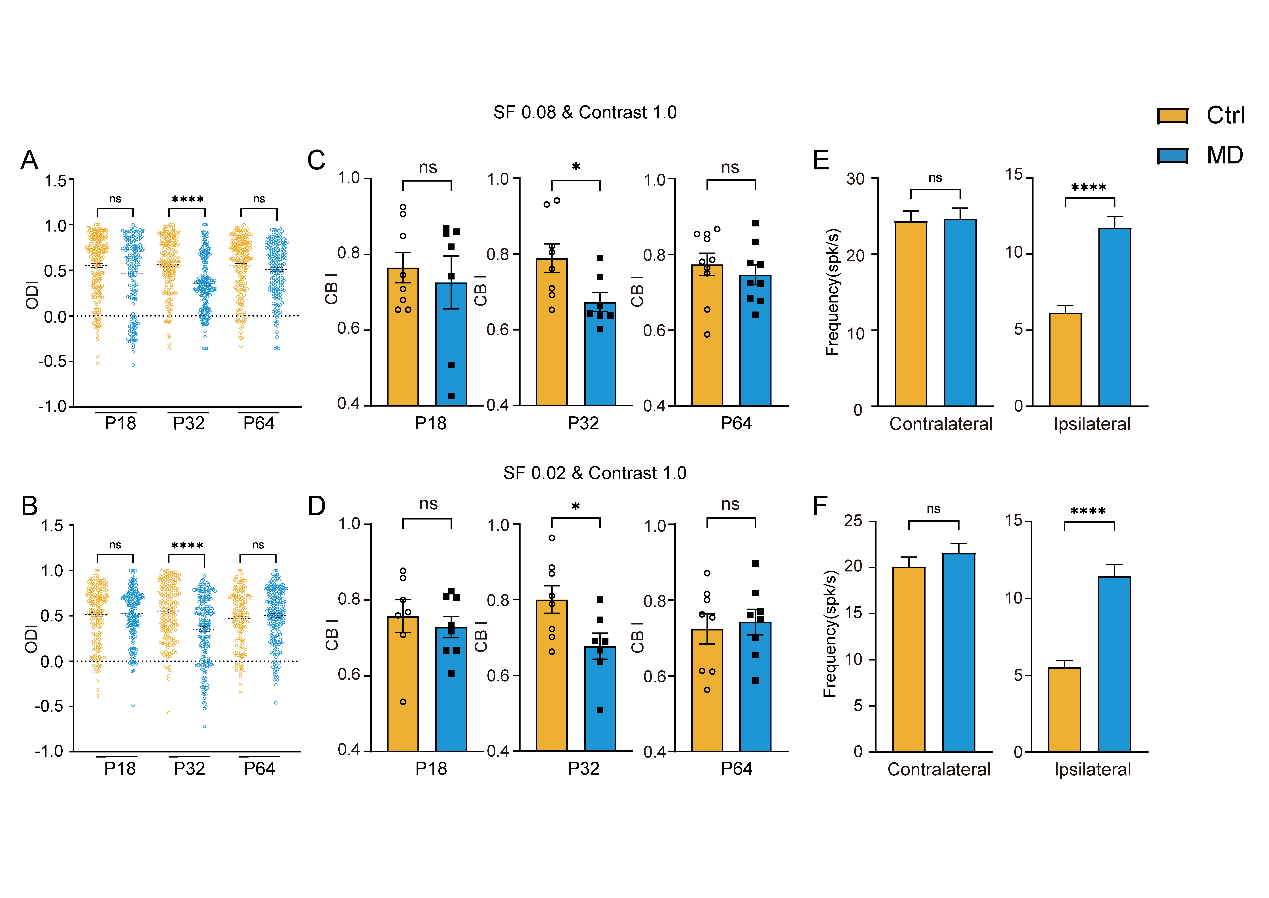
**

**Figure S5. MD induced OD shifts in dLGN neurons were independent of stimulus spatial frequencies.**

**A and B** Comparisons of ODI in P18, P32, and P64 between control (yellow) and MD group (blue) with SF 0.08 (A) and SF 0.02 (B) (Kruskal-Wallis test; for SF 0.08, P18 Ctrl, *n*=195 cells from 8 mice; P18 MD, *n*=154 cells from7 mice; P32 Ctrl, *n*=177 cells from 8 mice; P32 MD, *n*=214 cells from 7 mice; P64 Ctrl, *n*=185 cells from 10 mice P64 MD, *n*=180 cells from 9 mice; for SF 0.02, P18 Ctrl, *n*=168 cells from 7 mice; P18 MD, *n*=172 cells from 8 mice; P32 Ctrl, *n*=176 cells from 8 mice; P32 MD, *n*=189 cells from 7 mice; P64 Ctrl, *n*=138 cells from 8 mice; P64 MD, *n*=196 cells from 8 mice; respectively).ns *P* > 0.05, *****P* < 0.0001.

**C and D** Comparisons of CBI in P18 (left), P32 (middle), and P64 (right) between control and MD group with SF 0.08 (C) and SF 0.02 (D). Unpaired *t* test, two-tailed.ns *P* > 0.05, **P* <0.05.

**E and F** Comparisons of mean responses of contralateral (left) and ipsilateral eye (right) in dLGN neurons between P32 Ctrl and P32 MD mice in SF 0.08 (E) and in SF 0.02 (F). Mann-Whitney *U* test. Error bars represent mean ± SEM. ns *P* > 0.05, *****P* < 0.0001.

**
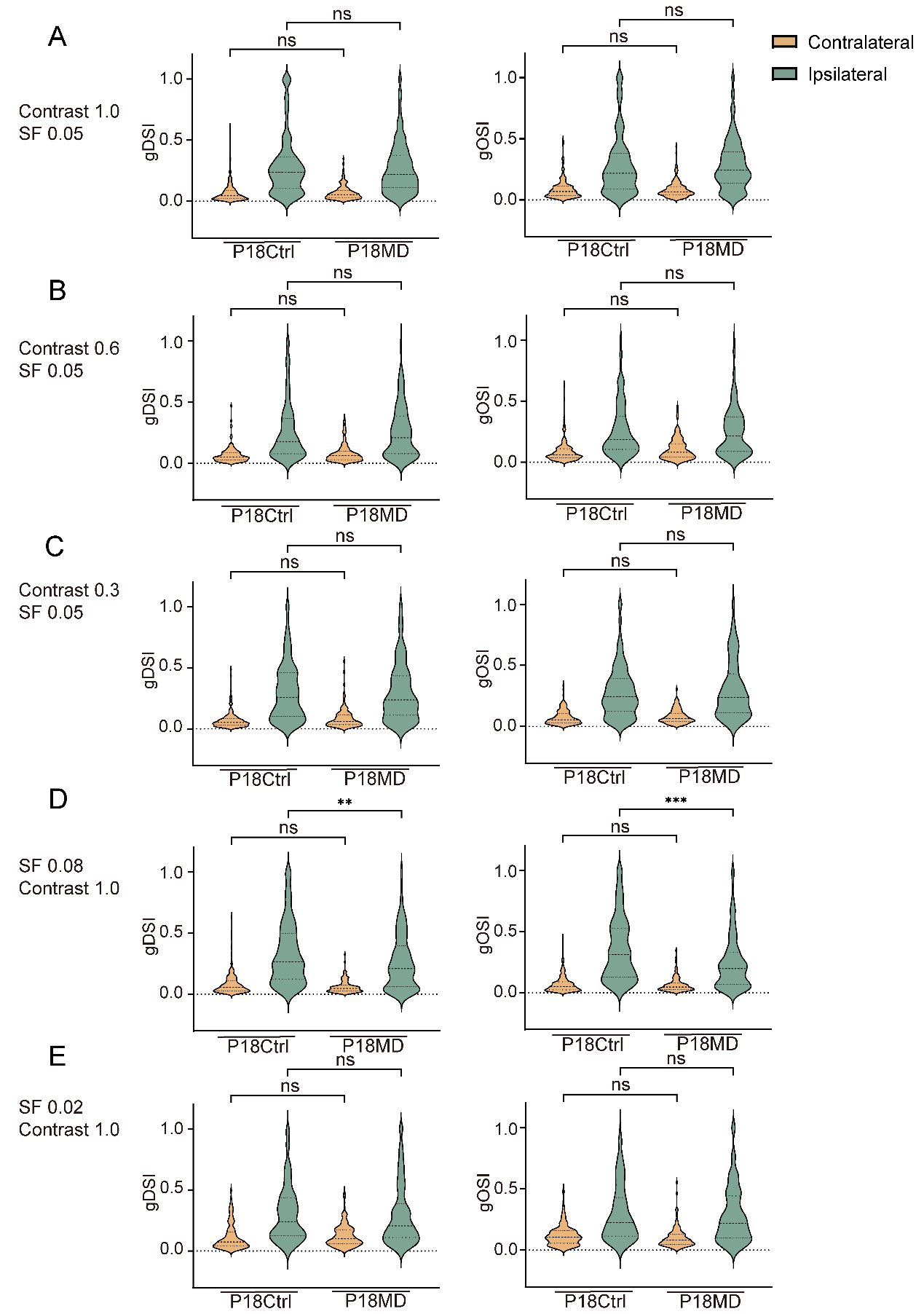
**

**Figure S6. 4-day MD during pre-critical period did not reduce DS and OS of ipsilateral eye responses under different contrast and SF conditions except for high SF.**

Comparisons of gDSI (left) and gOSI (right) of contralateral (yellow) and ipsilateral (green) eye responses in dLGN neurons between P18 Ctrl and P18 MD in contrast 1.0 /SF 0.05 (**A**; P18 Ctrl vs. P18 MD for gDSI, contralateral, *P* = 0.6914; ipsilateral, *P* > 0.9999; for gOSI, contralateral, *P* > 0.9999; ipsilateral, *P* = 0.4917; respectively), contrast 0.6 (**B**; P18 Ctrl vs. P18 MD for gDSI, contralateral, *P* = 0.2915; ipsilateral, *P* > 0.9999; for gOSI, contralateral, *P* = 0.0619; ipsilateral, *P* > 0.9999; respectively), contrast 0.3 (**C**; P18 Ctrl vs. P18 MD for gDSI, contralateral, *P* = 0.3174; ipsilateral, *P* > 0.9999; for gOSI, contralateral, *P* = 0.5295; ipsilateral, *P* > 0.9999; respectively), SF 0.08 (**D**; P18 Ctrl vs. P18 MD for gDSI, contralateral, *P* = 0.1999; ipsilateral, *P* = 0.0058; for gOSI, contralateral, *P* > 0.9999; ipsilateral, *P* = 0.0002; respectively), 0.02 SF (**E**; P18 Ctrl vs. P18MD for gDSI, contralateral, *P* = 0.1601; ipsilateral, *P* = 0.4454; for gOSI, contralateral, *P* = 0.0733; ipsilateral, *P* = 0.8024; respectively). A-E, Kruskal-Wallis test (for contrast 0.6, P18 Ctrl, *n*=177 cells from 7 mice; P18 MD, *n*=147 cells from 7 mice; for contrast 0.3, P18 Ctrl, *n*=169 cells from 7 mice; P18 MD, *n*=135 cells from 6 mice; for SF 0.08, P18 Ctrl, *n*=195 cells from 8 mice; P18 MD, *n*=154 cells from7 mice; for SF 0.02, P18 Ctrl, *n*=168 cells from 7 mice; P18 MD, *n*=172 cells from 8 mice), respectively. ns *P*>0.05, ** *P*<0.01 and *** *P*<0.001, respectively.

**
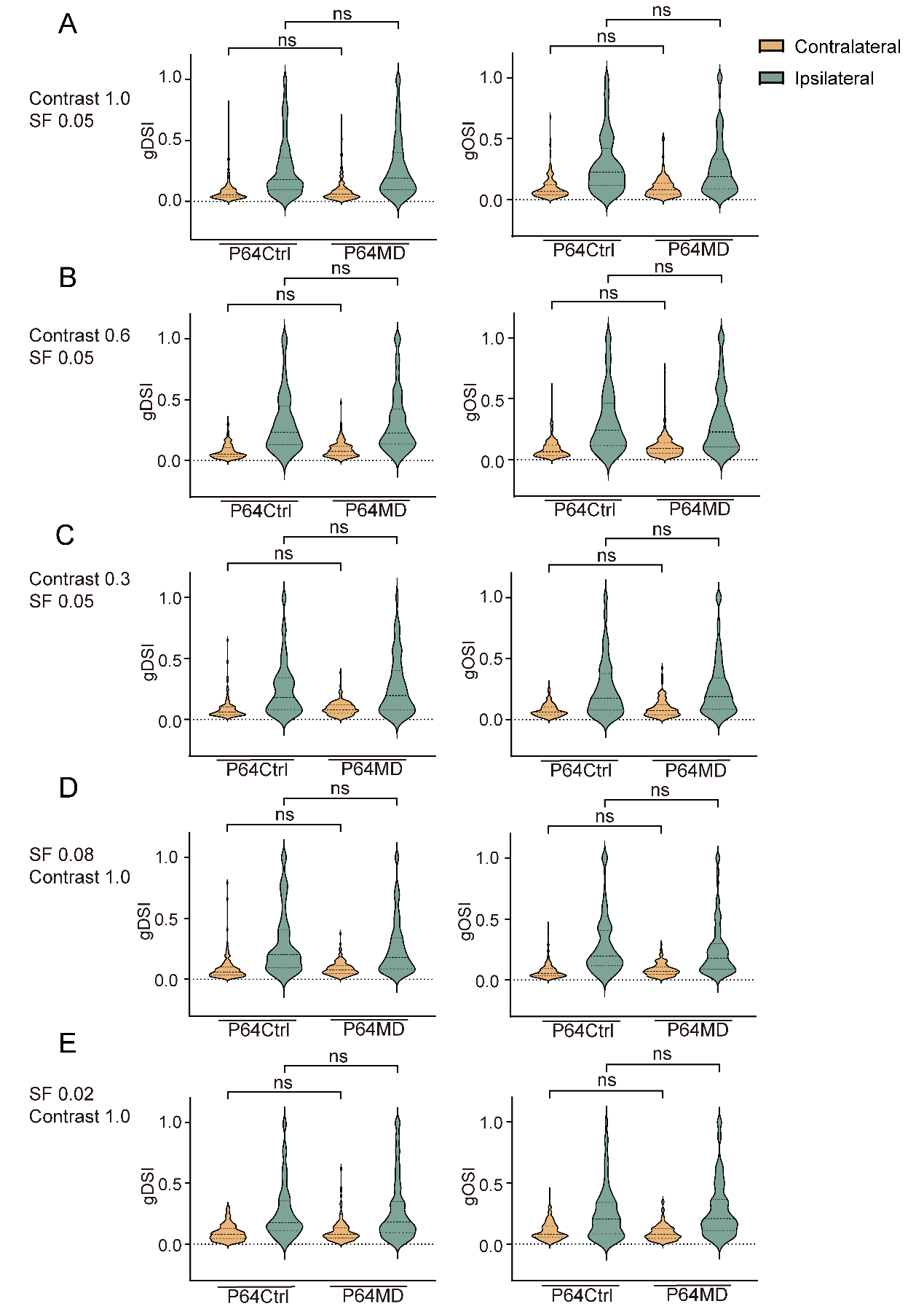
**

**Figure S7. 4-day MD during adulthood did not reduce DS and OS of ipsilateral eye responses under different contrast and SF conditions.**

Comparisons of gDSI (left) and gOSI (right) of contralateral (yellow) and ipsilateral (green) eye responses in dLGN neurons between P64 Ctrl and P64 MD in contrast 1.0 /SF 0.05 (**A**; P64 Ctrl vs. P64 MD for gDSI, contralateral, *P* = 0.9637; ipsilateral, *P*>0.9999; for gOSI, contralateral, *P* = 0.4541; ipsilateral, *P* = 0.1730; respectively), contrast 0.6 (**B**; P64 Ctrl vs. P64 MD for gDSI, contralateral, *P* = 0.1058; ipsilateral, *P* > 0.9999; for gOSI, contralateral, *P* = 0.0847; ipsilateral, *P* > 0.9999; respectively), contrast 0.3 (**C**; P64 Ctrl vs. P64 MD for gDSI, contralateral, *P* = 0.1406; ipsilateral, *P* > 0.9999; for gOSI, contralateral, *P* = 0.4249; ipsilateral, *P* = 0.9146; respectively), SF 0.08 (**D**; P64 Ctrl vs. P64 MD for gDSI, contralateral, *P* = 0.1805; ipsilateral, *P* = 0.6764; for gOSI, contralateral, *P* = 0.0797; ipsilateral, *P* = 0.5028; respectively), SF 0.02 (**E**; P64 Ctrl vs P64 MD for gDSI, contralateral, *P* > 0.9999; ipsilateral, *P* > 0.9999; for gOSI, contralateral, *P* = 0.4029; ipsilateral, *P* = 0.2825; respectively). A-E, Kruskal-Wallis test (for contrast 0.6, P64 Ctrl, *n*=161 cells from 8 mice; P64 MD, *n*=203 cells from 10 mice; for contrast 0.3, P64 Ctrl, *n*=165 cells from 8 mice; P64 MD, *n*=159 cells from 7 mice; for SF 0.08, P64 Ctrl, *n*=185 cells from 10 mice P64 MD, *n*=180 cells from 9 mice; for SF 0.02, P64 Ctrl, *n*=138 cells from 8 mice; P64 MD, *n*=196 cells from 8 mice), respectively. ns: *P*>0.05.
